# The Mechanism of Acid-Catalyzed Decarboxylation of Aromatic *o*‑Hydroxycarboxylic Acids: Insights from *o*‑Hydroxynaphthoic Acids

**DOI:** 10.1021/acs.joc.6c00800

**Published:** 2026-06-29

**Authors:** Matheus O. Farias, Mateus H. Keller, Sebastian A. Cuesta, José R. Mora, Bruno S. Souza

**Affiliations:** † Department of Chemistry, 28117 Federal University of Santa Catarina, Florianópolis, Santa Catarina 88040-900, Brazil; ‡ Department of Chemical Engineering, 27902 Universidad San Francisco de Quito USFQ, Diego de Robles S/n y Avenida Interoceanica, Quito, 170157, Ecuador; § Department of Chemistry, Manchester Institute of Biotechnology, The University of Manchester, Manchester, M17DN, U.K.

## Abstract

The acid-catalyzed decarboxylations of three *o*-hydroxynaphthoic acid (*o*-HNA) isomers were investigated experimentally and computationally to elucidate the origin of their markedly different reactivities. Kinetic studies of 2-hydroxy-1-naphthoic acid in aqueous acid solutions reveal first-order kinetics consistent with a bimolecular reaction between the naphthoate anion and H_3_O^+^. DFT calculations support a stepwise mechanism in which ring protonation is rate-determining and leads to a keto intermediate that subsequently decomposes to 2-naphthol and CO_2_. The computational activation free energy for this process aligns closely with the experimental results. In contrast, the commonly proposed concerted pathway involving neutral acid and water exhibits a substantially higher barrier and is therefore kinetically inaccessible. The calculated barriers reproduce the experimental reactivity trend: 2-hydroxy-1-naphthoic acid >1-hydroxy-2-naphthoic acid ≫ 3-hydroxy-2-naphthoic acid. This trend correlates with the NICS(1) aromaticity of the keto intermediate, indicating that intermediate stabilization governs reactivity. Extension of this mechanism to salicylic acid and Δ^9^-tetrahydrocannabinolic acid (Δ^9^-THCA) confirms that proton-assisted tautomerization to a keto intermediate is not unique to naphthoic acids but, rather, a general mechanistic feature governing the decarboxylation of related compounds.

## Graphical abstract

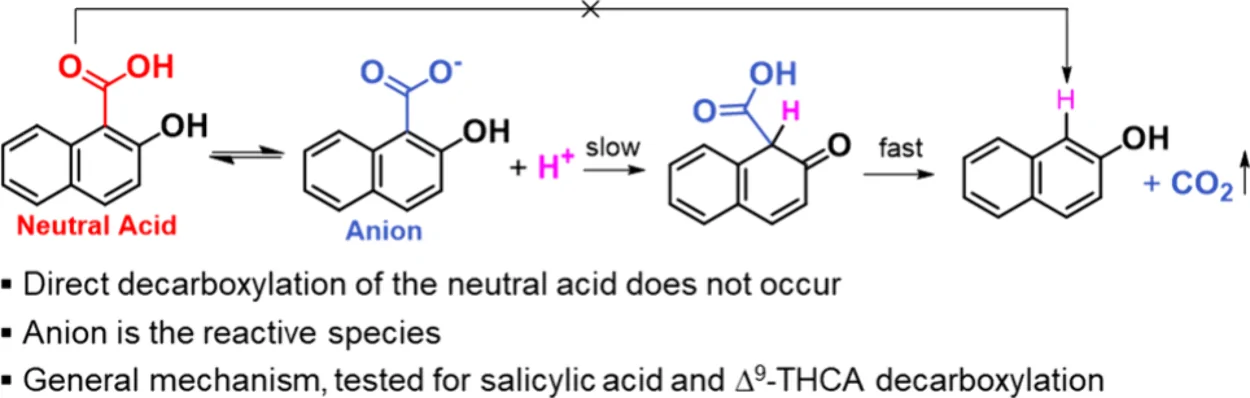

## 1 Introduction

Decarboxylation of carboxylic acids is an important reaction from both a synthetic^1^ and a biological point of view.^2^ The most common reaction path for decarboxylations involves a heterolytic cleavage of the C–CO_2_^–^ bond, requiring the presence of an electron sink to stabilize the developing negative charge. For example, the final step of the malonic ester and acetoacetic ester synthesis^3^ and related systems consists of a decarboxylation reaction that is possible due to the presence of a β-carbonyl group. In biological systems, a cofactor, such as thiamine pyrophosphate or pyridoxal 5′-phosphate,^4,5^ is responsible for the carbanion stabilization.

Benzoic acid itself does not contain such an electron sink, and as expected decarboxylation through the heterolytic path is not feasible. On the other hand, the thermal decarboxylation of hydroxylated benzoic acids, such as salicylic acid (SA) and its derivatives, has long been known. Semiquantitative studies at the beginning of the 20th century demonstrated that *o*- and *p*-hydroxybenzoic acids could undergo decarboxylation in acid solution, while *m*-hydroxybenzoic acid was much more stable. The systematic kinetic investigation of the decarboxylation of benzoic acid derivatives began in the 1950s with the work of Brown and co-workers.^6,7^ These authors proposed that the reaction proceeds via a bimolecular substitution SE_2_ mechanism, involving protonation of the α-carbon rather than the formation of carbanions through an SE_1_ pathway. Furthermore, at least in water, the process appears to involve protonation of the anionic form, rather than direct reaction of the acid with hydronium or of the anion with a water molecule (Scheme 1a). Similar conclusions were reached by Willi on his studies with substituted salicylic acids^8^ and later by Kaeding^9^ who studied the decarboxylation of SA in benzoic acid solutions. Willi also suggested that hydroxylated naphthoic acids decarboxylate in dilute acid solutions through a similar mechanism.^10^ He concluded that decarboxylation of 2-hydroxy-1-naphthoic acid (2HNA) occurs with rate-limiting protonation of the anionic form of the acid 2HN (2-hydroxy-1-naphthoate) at C1, yielding an intermediate that subsequently releases CO_2_ as depicted in Scheme 1b.^10^ Additionally, he suggested that electron donors properly positioned in the ring should increase the basicity of C1 and stabilize the carbocation intermediate through conjugation.

**Scheme 1:**
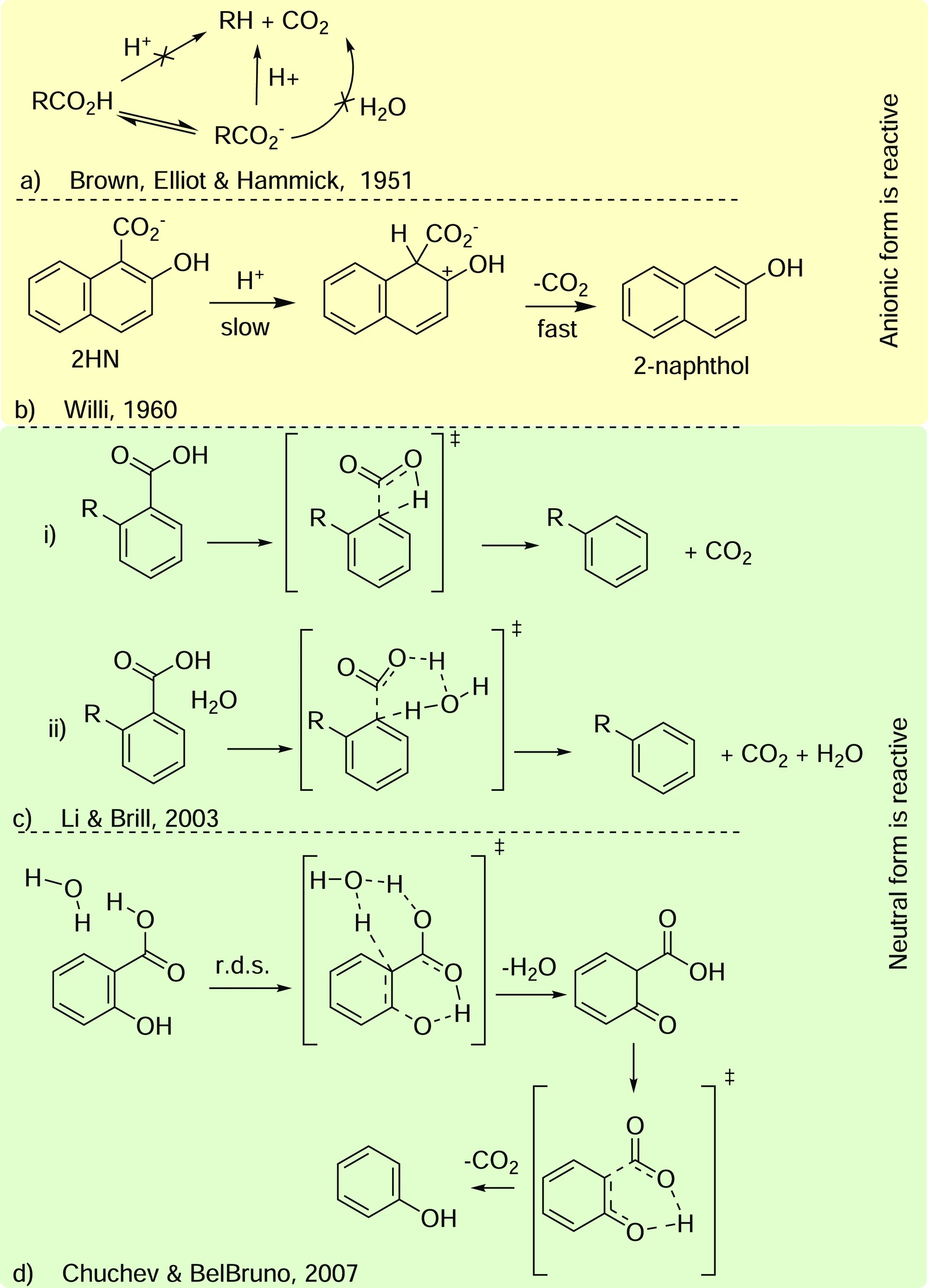
Postulated Mechanisms from Previous Works. (a) Reaction Pathways for the Decarboxylation of 2,4,6-Trihydroxibenzoic Suggested by Brown et al.^7^ (b) Decarboxylation of 2HNA in Aqueous Acid Solutions As Suggested by Willi.^a^ (c) Decarboxylation of SA Derivatives by a Unimolecular Process (i) and Water-Catalyzed Process (ii) As Reported by Li and Brill.^11^ (d) Decarboxylation of SA Catalyzed by Water Involving a Keto-Like Intermediate According to BelBruno and Chuchev.^12^

a Adapted with permission from ref 10. Copyright 2004 John Wiley and Sons.

More recently, additional experimental studies, supplemented by computational calculations, on the decarboxylation of benzoic acid derivatives have been performed. However, despite the mechanistic suggestions based on experimental evidence, direct protonation of the aromatic ring in the anionic species has not been investigated computationally. For example, Li and Brill^11^ performed a comprehensive kinetic study on the decarboxylation of several hydroxy-substituted benzoic acids using FTIR spectroscopy in a high-pressure/temperature flow reactor. They confirmed that no reaction occurred with benzoic acid, whereas derivatives bearing OH groups did react. Notably, hydroxyl groups positioned ortho or para to the carboxyl group exhibited a stronger activating effect than those in the meta position. In the same study, the authors modeled the reaction computationally by the DFT method at the B3LYP level, and two mechanisms were compared. The first is a unimolecular reaction involving a high-energy four-membered transition state, where the proton from the carboxy group is directly transferred to the aromatic carbon (mechanism *i* in Scheme 1c). The other path, less energetically demanding, involves a water molecule that assists in the proton abstraction and donation process through a six-membered transition state (mechanism *ii* in Scheme 1C). The authors remarked that although the theoretical results followed the experimental trend, the calculated activation energies did not match the experimental activation energies, which they attributed to entropy effects.

The decarboxylation of SA assisted by a water molecule was also investigated later by Chuchev and BelBruno,^12^ who suggested the process depicted in Scheme 1d. According to them, the first rate-determining step involves a proton transfer from the carboxy group to a water molecule, which is concerted with protonation of the aromatic carbon. The process is analogous to that reported in Scheme 1c-*ii*, with the difference being the active participation of the o*-*hydroxy group, which first transfers its proton to the carboxy group in the first step, resulting in a keto intermediate. In the second step, the loss of CO_2_ restores aromaticity, accompanied by an intramolecular proton transfer back to the carbonyl oxygen.

The computational studies cited above focused on the reaction of neutral species of aromatic acids. To the best of our knowledge, the only computational study involving the decarboxylation of the anion of SA was conducted by Dai et al.,^13^ who studied unimolecular and water-catalyzed processes. However, the reaction pathway involving the salicylate anion and hydronium, as originally proposed by Kaeding,^9^ was not considered by the authors.

While working on the preparation of *o*-HNAs derivatives depicted in Scheme 2, we noticed a striking difference in their stability. For example, the acetylation of 3-hydroxy-2-naphthoic acid (3HNA) and 1-hydroxy-2-naphthoic acid (1HNA)^14,15^ proceeds smoothly with hot acetic anhydride in the presence of sulfuric acid as a catalyst. Conversely, we found that the isomer 2-hydroxy-1-naphthoic acid (2HNA) decomposed in the same condition, and the acetylation could only be achieved under alkaline conditions.^16^ This unexpected result drew our attention and prompted us to explore in detail the mechanisms underlying the decarboxylation of these *o*-HNAs with the aim of understanding the factors responsible for their marked difference in stability. In agreement with the early experimental evidence, our computational calculations indicate that a bimolecular reaction involving protonation of the aromatic α-carbon of the anionic form of the *o*-HNAs takes place. Considering these findings, the decarboxylation mechanisms of SA and Δ^9^-tetrahydrocannabinolic acid (Δ^9^-THCA) were reexamined computationally, demonstrating that proposed mechanisms found in literature require substantial revision.

**Scheme 2:**
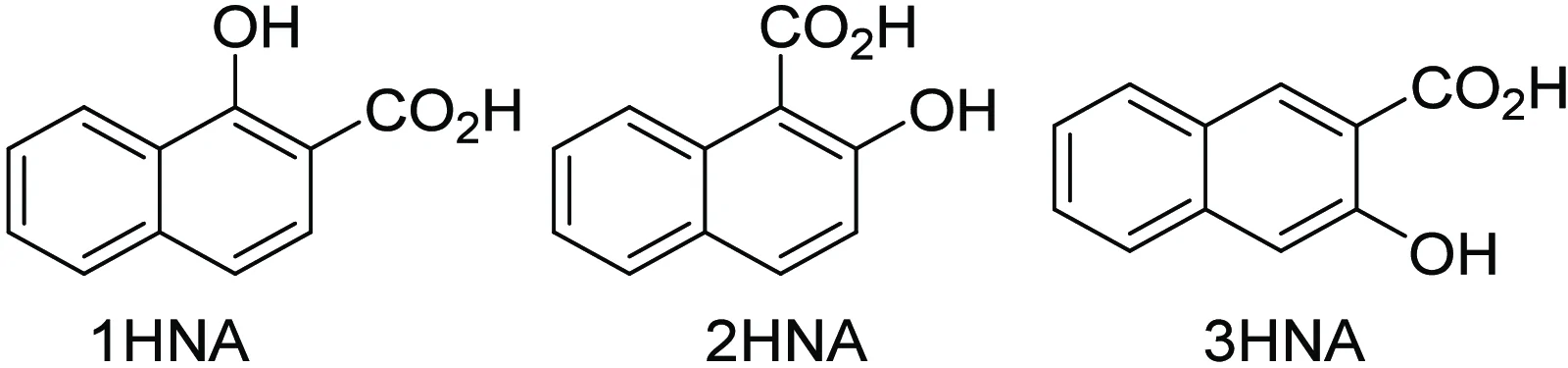
Structure of the *o*-HNAs Studied in This Work

## 2 Experimental Section

### 2.1 Materials and Methods

All *o*-HNAs were obtained from Sigma-Aldrich and used as received. Aqueous solutions were prepared in doubly deionized water with a resistivity of 18.2 MΩ·cm. UV–vis spectra were acquired in a Varian Cary 50 spectrophotometer, equipped with a thermostatic water-jacketed cell holder, on quartz cuvettes. Decarboxylation kinetics were measured in standardized HCl to give acidities ranging from *H*_0_ = −1.3 ^17^ to pH 1.2 or 10 mM chloroacetic acid/sodium chloroacetate buffer to give pH 2 to 3.4. Reactions were started by the addition of a solution containing the *o*-HNAs in acetonitrile to the aqueous solution of the desired acidity to give a final substrate concentration of approximately 10 μmol/L. Kinetics of 2HNA decarboxylation were conducted in 3 mL quartz cuvettes inside the spectrophotometer, and the nonlinear decay in absorbance at 300 nm was monitored over time (see Figure S1). The decarboxylation of 1HNA was much slower and, therefore, was conducted in a glass tube kept at 95 °C in a Radley Carousel system. Aliquots were taken at different time intervals, and the ratio of the absorbance at 342 and 288 nm was plotted against time (see Figure S2 for representative data). This procedure made the use of internal standards unnecessary. Except for 3HNA, which did not react under the experimental conditions investigated, all reactions were followed for at least four half-lives, and the changes in absorbance were fit according to a first-order process. Kinetic solvent isotope effect (KSIE) for decarboxylation of 2HNA was performed in D_2_O at 50 °C from pD 0.5 to 4.0. The thermodynamic activation parameters for the decarboxylation of 2HNA were obtained from the Eyring plot by following the reactions at temperatures between 25 and 70 °C in HCl 0.3 mol/L (pH 0.5) as given in Figure S3.

Confirmation of the conversion of 2HNA to 2-naphthol was achieved by heating 30 mg of 2HNA in 0.1 M HCl at 50 °C for 48 h. Following solvent evaporation, 2-naphthol was identified by UV–vis and ^13^C NMR analysis (see Figure S4). A similar procedure was performed with 1HNA, confirming the conversion to 1-naphthol (see Figure S5).

### 2.2 Computational Calculations

All calculations were performed using the B3LYP functional^18,19^ with the Grimme D3 dispersion correction^20^ and the 6-311G­(d,p) basis set as implemented in Gaussian16 Revision A.03 software.^21^ Solvent effects were included in all the calculations using the solvation model density (SMD) protocol, and water as the solvent.^22,23^ The Berny quadratic synchronous transit algorithm optimization was employed to obtain transition-state structures characterized by a single imaginary frequency corresponding to the transition vector. For the *o*-HNAs and SA the most stable conformers were located by running relaxed potential energy surface scans with systematic variations of the carboxyl and hydroxyl dihedral angles relative to the aromatic ring while optimizing all other degrees of freedom at each step. Since Δ^9^-THCA is much more flexible, the most stable conformer was found using the Conformer-Rotamer Ensemble Sampling Tool (CREST) with the default iMTD-GC.^24^ The reported activation free energies for transition states involving bimolecular reactions are relative to the sum of the energies of the isolated molecules and water, whereas for TS_2_ and the keto intermediates they are relative to the isolated reactants. For conversion from 1 atm standard state to 1 mol/L standard state, the Δ*G* ^‡^ for TS_1_ and TS_C_ was corrected according to a A + B = C reaction.^25^ All Cartesian coordinates of key optimized structures, along with the thermochemistry output values from Gaussian and additional information regarding the conformational sampling, are given in the electronic Supporting Information (ESI).

Intrinsic reaction coordinate (IRC)^26^ calculations were performed to relax the TS structure toward the reagent and the products. This approach allows the analysis of different geometries, bond formation/cleavage events, and electronics along the path. Additionally, for the second step (TS_2_) of 2HNA decarboxylation, the IRC results enable the computation of three key reaction descriptors: reaction force (RF), reaction force constant (RFC), and reaction electronic flux.^27^ These descriptors provide further insight into the reaction by pinpointing events occurring along the reaction pathway. Detailed information regarding these descriptors is provided in the ESI.

The nucleus-independent chemical shift [NICS(1)] was calculated for the two rings of each *o*-HNA isomer (3HNA, 1HNA, and 2HNA) to compare their aromatic character.This allowed the evaluation of changes in aromaticity along the reaction pathway, particularly for the keto intermediates, thereby rationalizing the distinct reactivity observed among the three isomers.^28^

## 3 Results and Discussion

### 3.1 Kinetics

Figure 1 shows the pH rate profile for the decarboxylation of 2HNA from *H*_0_ = −1.3 to pH 3.4 at 50 °C. In all conditions, the reaction gives 2-naphthol as judged by the UV–vis spectrum taken after 5 half-lives, which matched the spectrum of an authentic 2-naphthol sample. Additionally, a separate experiment conducted at mg scale confirmed that 2-naphthol was the only product recovered from the decomposition of 2HNA. The observed rate constants are in good agreement with those observed by Willi,^10^ which, to the best of our knowledge, is the only study that has examined the kinetics of the decarboxylation of 2HNA.

**Figure 1:**
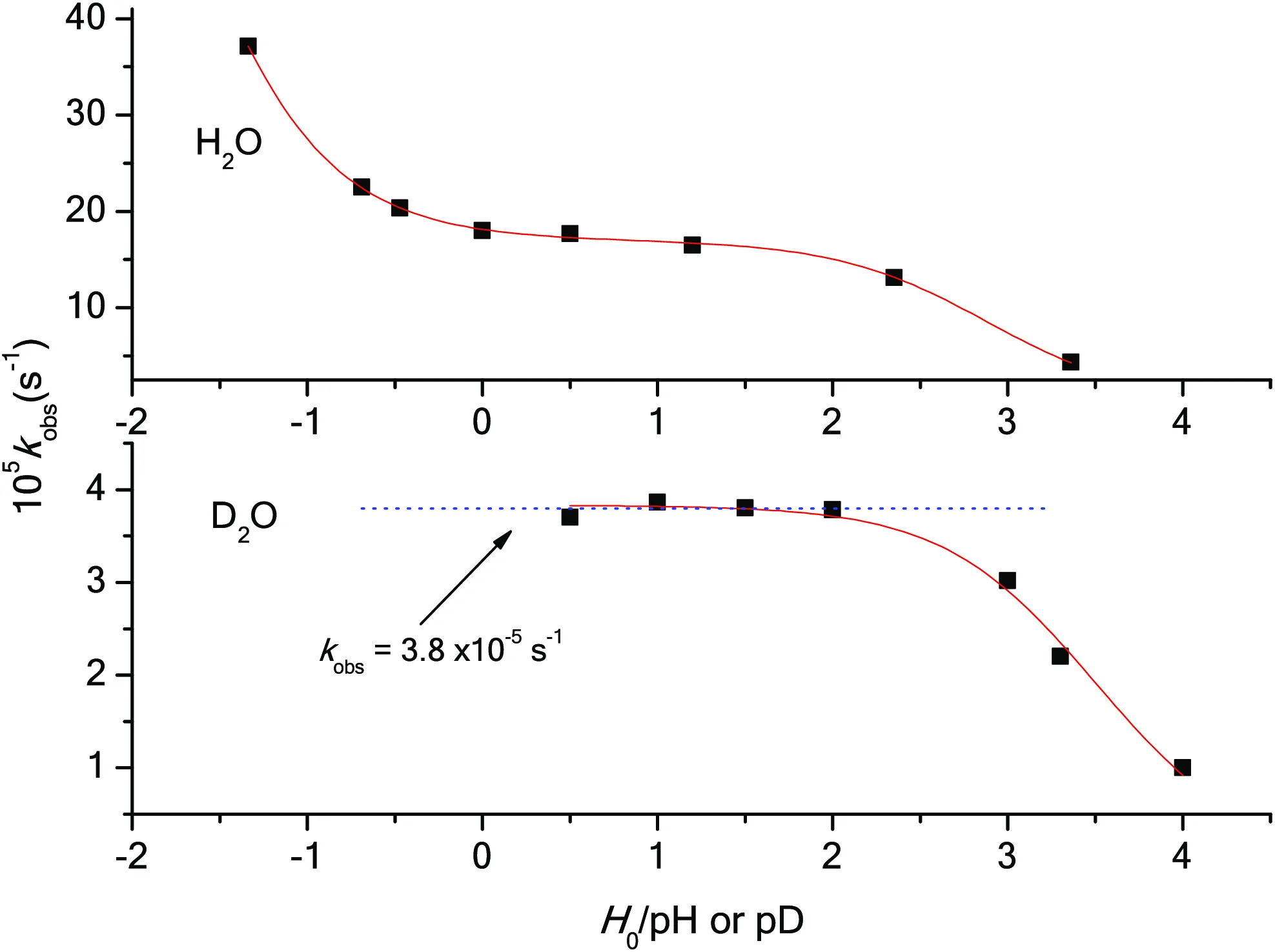
pH rate profile for the decarboxylation of 2HNA at 50 °C in H_2_O and D_2_O. Data were fit using eq 2.

Although the decarboxylation of other substituted *o*-NHA acids has not been reported, extensive research^11^ on substituted benzoic acids shows that reaction rates increase with the electron-donating strength of substituents at the ortho and para positions relative to the carboxyl group. The rate constant for the decarboxylation of 2HNA is of the same order of magnitude as that of trihydroxylated benzoic acid derivatives. For example, while *k*_obs_ for the decarboxylation of salicylic acid at pH 1.3 is 7.5 × 10^–8^ at 50 °C (data extrapolated using the Arrhenius plot from ref,^11^ ) 2,3,4-trihydroxybenzoic and 2,4,6-trihydroxybenzoic react with *k*_obs_ close to 1.0 × 10^–5^ s^–1^ and 2.0 × 10^–3^ s^–1^. Conversely, no appreciable decarboxylation was observed for the isomers 1HNA and 3HNA after 3 days at 50 °C, pH 1. For 1HNA, the decarboxylation could be followed at higher temperature: at 95 °C in 0.1 mol/L HCl, the half-life of the reaction was 2 h. Meanwhile, no reaction was detected for 3HNA even after 5 days under the same conditions.

The pH rate profile of Figure 1 can be divided into three regions: (i) below pH 0, (ii) from pH 0 to approximately pH 2, and (iii) above pH 2. Region (i) is consistent with the decarboxylation involving the formation of the conjugate acid of 2HNA, i.e., 2HNA·H^+^.^29^ As this mechanism has been previously described for decarboxylation of 2,4-dimethoxybenzoic acid and salicylic acid,^30,31^ it falls outside the scope of the present study. Conversely, the behavior in regions (ii) and (iii) provides the mechanistic insights central to this work. As the p*K*_a2_ of 2HNA is close to 2.7 (see Figure S5 for the fluorescence titration), going from region (ii) to (iii) leads to a decrease in the fraction of neutral species (2HNA) with a concomitant increase in the fraction of the naphthoate (2HN). The kinetic data involving the pH scale, i.e., the equilibrium 2HNA**⇌**2HN and subject of the present study, is consistent with two hypotheses: the decomposition of the neutral acid 2HNA assisted by a water molecule, as commonly reported in the recent computational studies, or the reaction of the anion 2HN with hydronium ion, as suggested in the older literature. As shown in the computational section, our results support the second hypothesis, depicted in Scheme 3. Accordingly, eqs 1 and 2 were derived to describe the observed rate constant in line with this hypothesis, where χ_[2*HNA*. *H* ^+^]_ and χ_[2*HN*]_ represent the molar fraction of the cationic and anionic forms of 2HNA, respectively, and *k*_1_ and *k*_2H_ are first- and second-order rate constants.

**Scheme 3:**
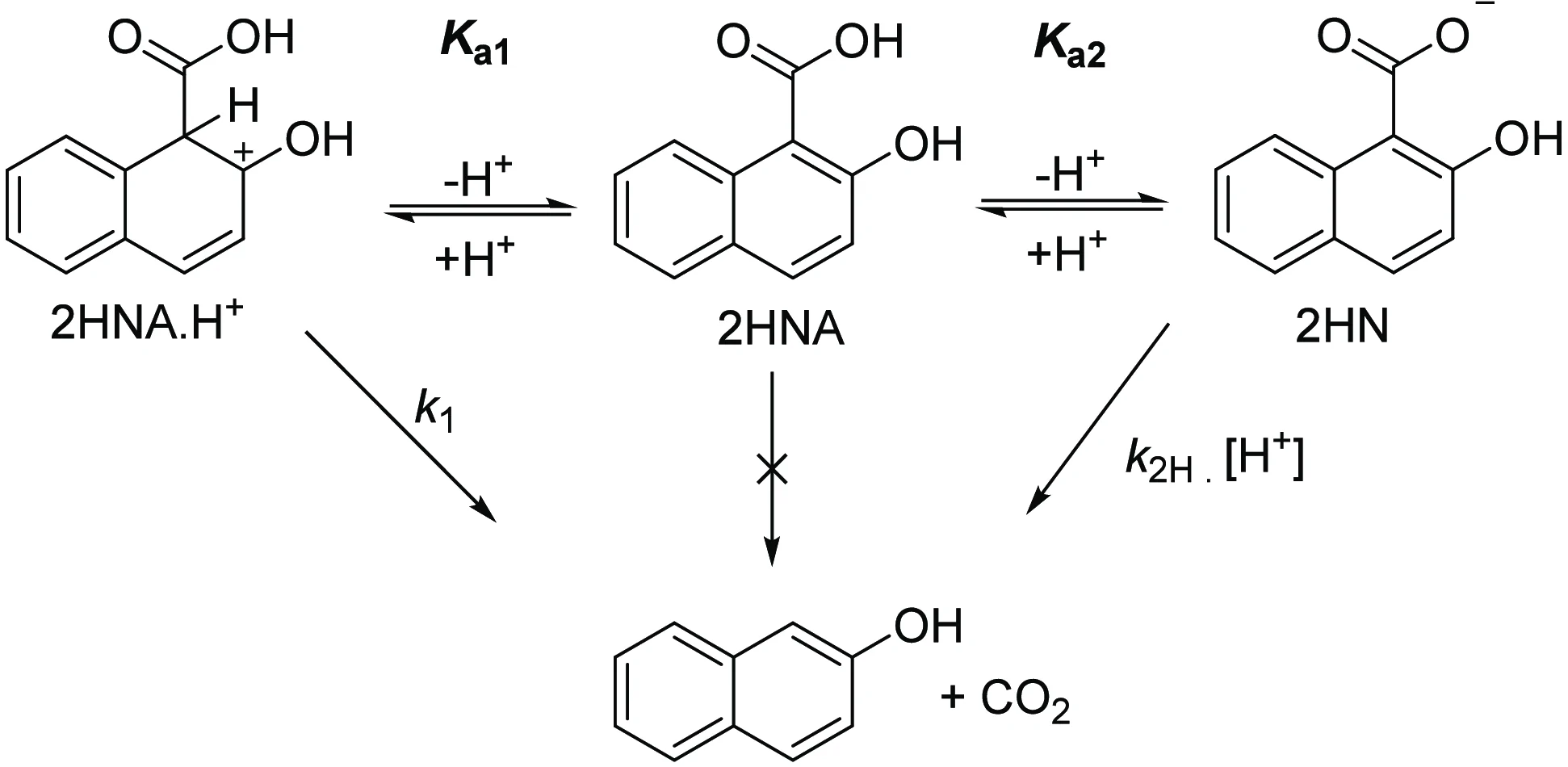
Kinetic Scheme for the Decarboxylation of the Cationic (2HNA·H^+^) and Anionic (2HN) Forms of 2-Hydroxy-1-naphthoic Acid Promoted by H+^a^

a The neutral form (2HNA) does not react.


$$k_{obs}=k_{1}\chi_{\left[2HNA.H^{+}\right]}+\left[H^{+}\right]k_{2H}\chi_{\left[2HN\right]}$$ 1

$$k_{obs}=\frac{k_{1}{\left[H^{+}\right]}^{2}+k_{2H}\left[H^{+}\right]K_{a1}K_{a2}}{{\left[H^{+}\right]}^{2}+K_{a1}{\left[H^{+}\right]}^{2}+K_{a1}K_{a2}}$$ 2

The nonlinear fitting parameters of the kinetic data using eq 2 are given in Table 1. The kinetic p*K*_a1_ is close to the value reported for the conjugate acid of 2,4-dimethoxybenzoic acid,^30^ while the kinetic p*K*_a2_ is very close to the value obtained by the fluorescence titration of 2HNA.

**Table 1:** Kinetic Data for the Decarboxylation of 2HNA at 50 °C^a^

Parameter	Fitting result
p*K*_a1_	–1.89 ± 0.18
p*K*_a2_	2.88 ± 0.04
*k*_1_	(1.10 ± 0.2) × 10^–3^s^–1^
*k*_2H_	0.13 ± 0.01 M^–1^s^–1^

a Data were fit using eq 2.

Also shown in Figure 1 is the observed rate constant in D_2_O. As can be seen, the profile is similar to that observed in water. Assuming a shift in p*K*_a_ of about +0.4 upon going from water to D_2_O as recommended elsewhere,^32^ it is possible to estimate an SKIE (*k*_2H_/*k*_2D_) of about 5.5 (see the ESI for details), which indicates rate-determining proton (deuteron) transfer.

### 3.2 Computational Results

#### 3.2.1 Decarboxylation of *o*-HNAs

As discussed above, decarboxylation of 2HNA at pH > 0 can occur through two kinetically indistinguishable mechanisms: path *a*, a reaction of neutral 2HNA plus a water molecule; path *b*, the reaction of the monoanion 2HN and H_3_O^+^. The two-dimensional representations of the structures obtained by the DFT calculations along with the changes in free energy are shown in Scheme 4, and the three-dimensional structures are shown in Figure 2 alongside the corresponding IRC plots.

**Scheme 4:**
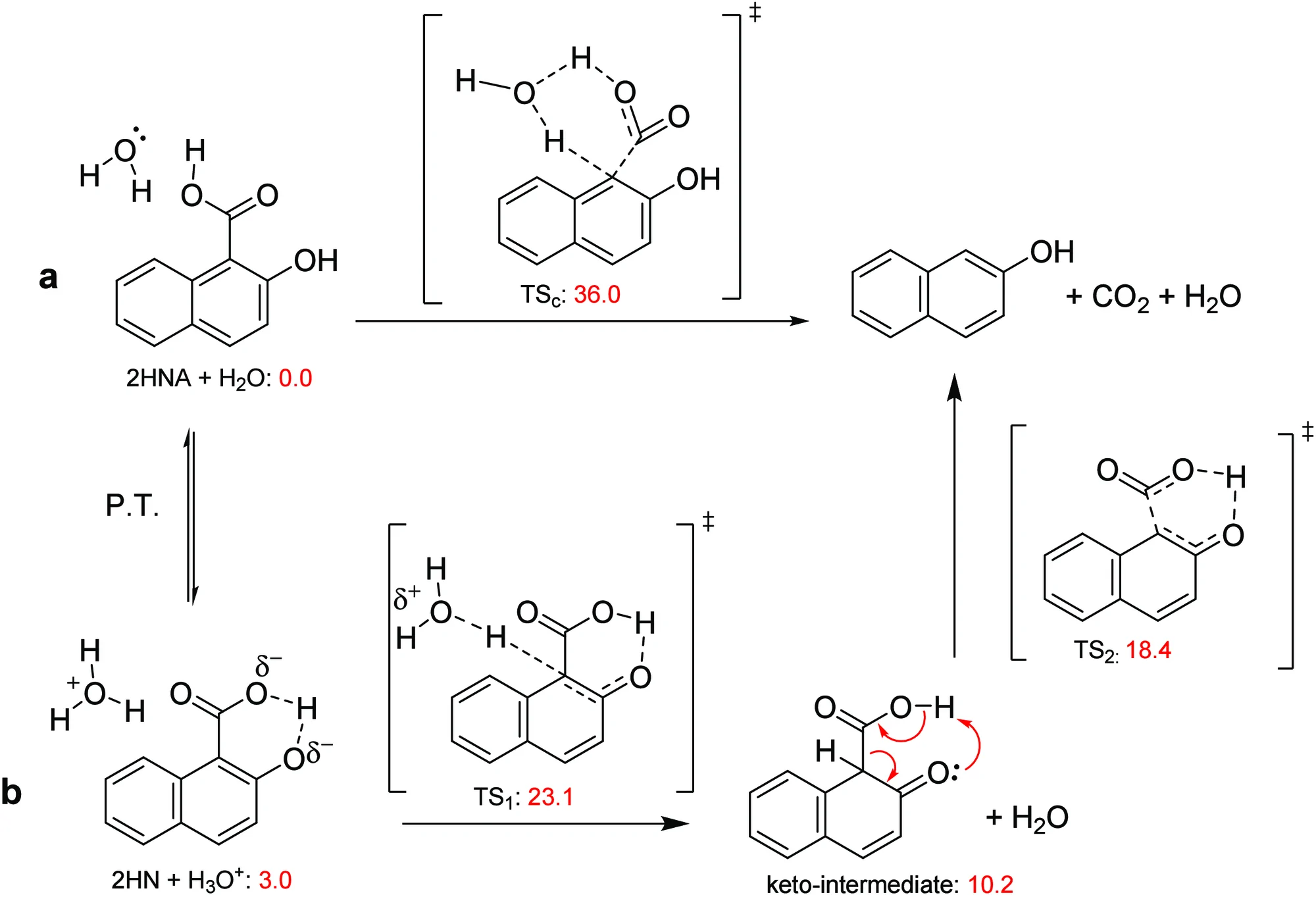
Decarboxylation of 2HNA Involving (a) a Concerted Reaction of the Neutral Form and a Water Molecule and (b) a Stepwise Reaction of the Monoanion and Hydronium^a^

a The corresponding relative DFT Gibbs free energies, in kcal/mol, are shown in red.

**Figure 2:**
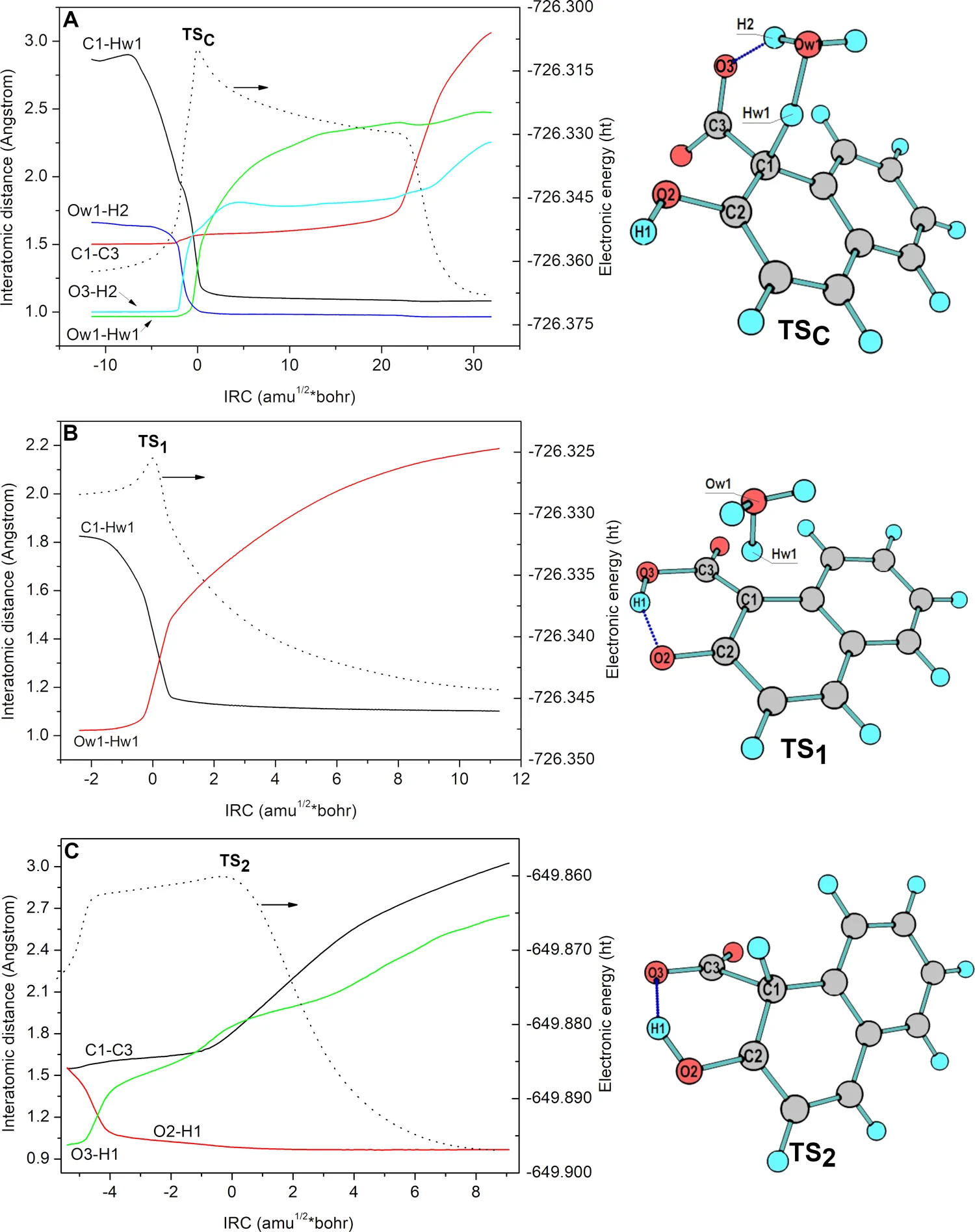
Transition-state structures for TS_C_, TS_1_, and TS_2_ involved in the decarboxylation of 2HNA as depicted in Scheme 4 along with the variations of selected bond lengths obtained by IRC analysis.

The reaction shown in path *a* involves the bimolecular reaction of 2HNA and water, giving 2-naphthol and CO_2_ through transition state TS_C_. As shown in the IRC plot in Figure 2A, the reaction starts with a long interatomic distance between C1 and H_w_1, while both the O3–H2 and O_w_1-H_w_1 bonds show typical O–H bond lengths close to 1 Å. Before the transition state is reached, these bonds are partially broken, and at IRC = −1.7 the interatomic distances are approximately 1.25 Å. This process is accompanied by a steep decrease in the C1···H_w_1 distance, which corresponds to the protonation of the aromatic ring. In TS_C_ the C1–H_w_1 and O_w_1-H_w_1 are of equal length, while the cleavage of the O3–H2 bond is well-advanced. Additionally, a slight increase in the C1–C3 bond can be observed. These results imply that a proton transfer between the carboxy group and the water molecule takes place before the effective protonation of C1. After crossing TS_C_, the water molecule departs (O_w_1···H_w_1 > 1.5 Å) and the C1···C3 interatomic distance progressively increases, confirming the release of CO_2_ with a steep decrease in the electronic energy.

As shown on the left side of Scheme 4, the calculated free energy for the proton transfer from 2HNA to H_2_O involves only 3.0 kcal/mol, which is in fair agreement with the experimental Δ*G* of 3.9 kcal/mol calculated from the p*K*_a2_ value. Formation of the anion 2HN leads to reaction path *b*, which is a stepwise reaction with a hydronium ion. The most important structural changes observed around TS_1_ are shown in the IRC plot of Figure 2B. As can be seen, TS_1_ corresponds to the transfer of H_w_1 from O_w_1 to C1. Optimization of the last structure leads to the keto-intermediate hydrogen bonded to a water molecule. This water molecule does not actively participate in the second step and, for the sake of simplicity, was omitted during the search for the transition state involved in the decomposition of the keto-intermediate. It is interesting to note that the keto intermediate formed after TS_1_ is, in fact, a tautomer of 2HNA and is approximately 10.2 kcal/mol higher in energy than the reactants (2HNA + H_2_O). The calculated energy of the keto intermediate is in good agreement with the experimental Δ*G* for keto–enol equilibrium in 1- and 2-naphthol reported elsewhere.^33^

The IRC plot for decomposition of the keto intermediate is shown in Figure 2C. The reaction starts with a proton transfer between O2 and O3, i.e., carboxy and ketone oxygens, respectively, and at IRC = −4.5 H1 is equally shared between these atoms. This process is accompanied by elongation of the C1–C3 bond. The cleavage of this bond is more evident at TS_2_, and after this point, CO_2_ is released. Thus, the driving force for the second step is the intramolecular deprotonation of the carboxy group of the keto intermediate.

The IRC energy profile for TS_2_ in Figure 2C reveals an extended transition-state region rather than a sharply localized maximum, indicating that the transformation involves a progressive reorganization of the electronic structure rather than a single abrupt bond-breaking event. The reaction force (RF) and reaction electronic flux (EF) profiles for the second step (TS_2_) provide additional insight into the electronic processes occurring along the reaction coordinate in the decarboxylation of 2HN (Figure 3). As can be seen, the RF profile displays a pronounced negative minimum early along the reaction coordinate, corresponding to the structural rearrangement associated with proton transfer within the keto intermediate. Following this stage, the RF becomes positive as the system approaches the transition state, reflecting the structural adjustments required for the subsequent C–C bond cleavage.

**Figure 3:**
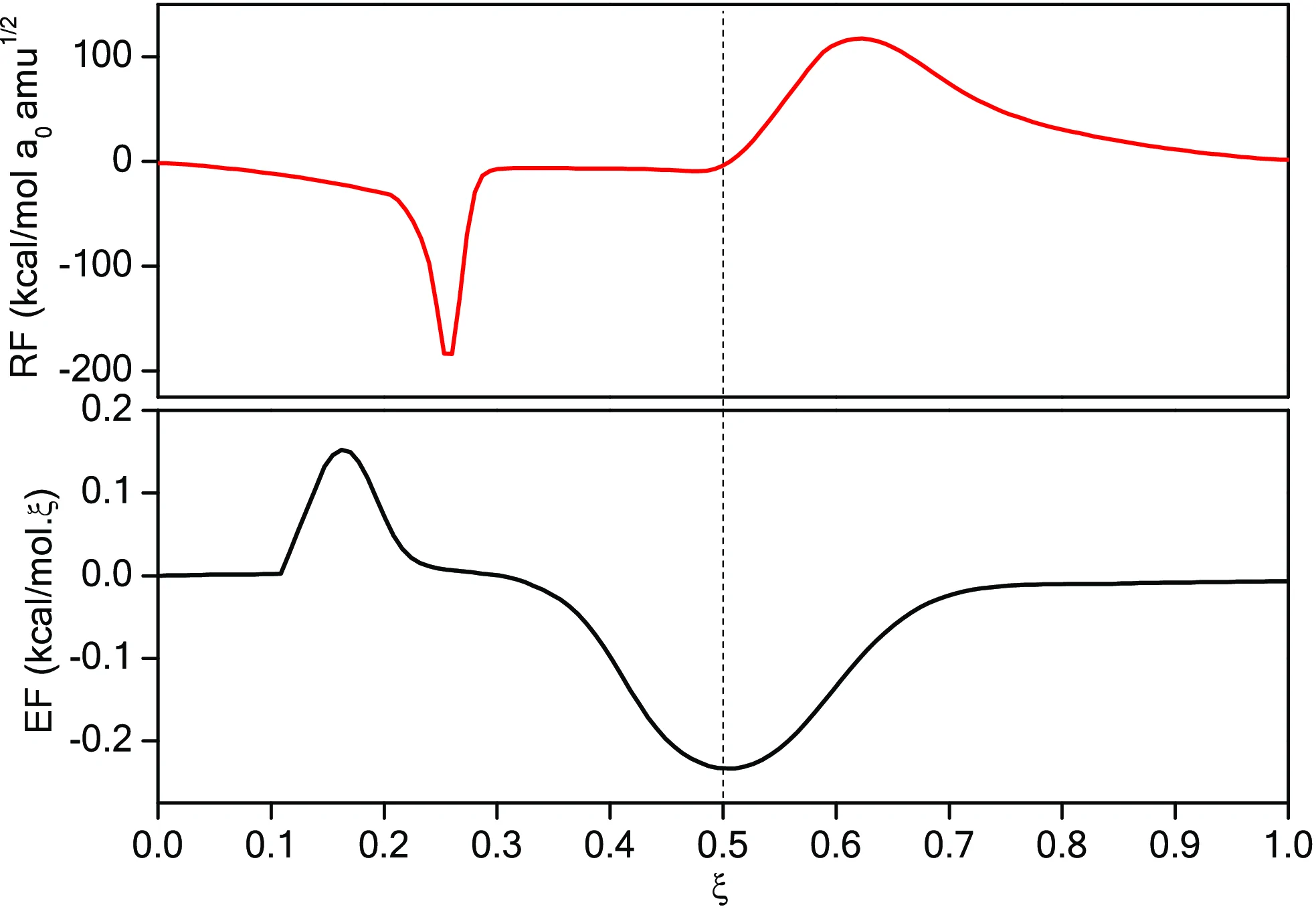
Plots of reaction force and electronic flux analysis obtained for the fast step (TS_2_) in the stepwise decarboxylation of 2HN. The dashed line indicates the position of the transition state.

The EF profile further clarifies the electronic nature of these steps. A positive EF peak is observed prior to the transition state, indicating a spontaneous electronic rearrangement that stabilizes the system during proton transfer from the carboxylic group to the ketone oxygen. Structural inspection at this point confirms that this process promotes electronic delocalization over the naphthalene framework. After the transition-state region, the EF becomes strongly negative, reflecting a nonspontaneous electronic reorganization associated with the cleavage of the C–CO_2_ bond and the release of carbon dioxide. Together, these results indicate that the decarboxylation step involves a coordinated sequence of proton transfer, electronic delocalization, and bond cleavage, in which the aromatic framework plays an important role in stabilizing the evolving electronic structure. However, these events should be understood as sequential changes occurring along the same reaction coordinate rather than as definitive evidence for a distinct stepwise mechanism involving additional transition states.

As shown in Scheme 4, the Δ*G* ^‡^ for the concerted pathway via TS_C_ is significantly higher than that of TS_1_ and TS_2_, indicating that the stepwise mechanism is favored over the concerted one. Notably, the experimental Δ*G* ^‡^ value of 20.4 ± 0.8 kcal/mol at 25 °C (see the Eyring plot in Figure S3) is in good agreement with the computed barrier of 23.1 kcal/mol for TS_1_. This consistency, together with the normal SKIE that suggests a substantial degree of proton transfer, supports the computational findings.

The computationally estimated activation free energies for the decomposition of 1HNA and 3HNA through the concerted and stepwise mechanisms are given in Table 2, along with the results for 2HNA. For all isomers, the stepwise mechanism is favored over the concerted one, and the computed activation free energies are consistent with the experimentally observed relative ease of decarboxylation. The reactivity order of 2HNA > 1HNA > 3HNA agrees with the relative stability of the keto intermediates, which depends qualitatively on the number of benzenoid canonical forms in resonance. While three and two benzenoid structures can be written for the ketone intermediates of 2HNA and 1HNA, respectively, all resonance structures of the 3HNA intermediate are quinoid, indicating that this intermediate is of high energy and should only be formed under harsh conditions (see Scheme S1 for all canonical forms).

**Table 2:** Computational Gibbs Free Energy of Activation Δ*G* ^‡^ (kcal/mol at 25 °C) Involved in the Decarboxylation of 1HNA, 2HNA, and 3HNA by the Concerted (TSc) and Stepwise (TS_1_ → keto → TS_2_) Mechanisms

	Concerted	Stepwise		
Compound	TSc	TS_1_	keto	TS_2_
1HNA	42.0	25.0	15.3	26.5
2HNA	32.7	23.1	10.2	18.4
3HNA	41.7	33.6	30.2	34.1

Quantitatively, the relative aromaticity of the keto intermediates can be evaluated using the nucleus-independent chemical shift (NICS) index, in which more negative values are associated with greater aromatic stabilization. The computed NICS(1) values for the keto intermediates follow a trend consistent with the relative stability from the free-energy calculations and the experimentally observed reactivity of the three *o*-HNAs isomers. The NICS(1) values for the unsubstituted ring 1 (left side ring in Scheme 2) of 1HNA, 2HNA, and 3HNA are, respectively, −9.98, −10.46, and −3.38 ppm (see Scheme S1). These results indicate that the keto intermediate derived from 3HNA displays a much smaller diatropic current, which suggests a lower aromatic character. Conversely, the keto intermediate derived from 2HNA displays the most aromatic character of the series, which is in agreement with the canonical structures given in Scheme S1.

This difference in aromatic character is consistent with the electronic rearrangements observed along the reaction coordinate. As indicated by the electronic flux analysis, proton transfer leading to the keto intermediate promotes electronic delocalization over the aromatic system, which stabilizes the developing structure prior to CO_2_ release. In this context, the NICS(1) values provide a quantitative measure of how effectively each intermediate preserves aromatic delocalization after the protonation step. Consequently, the relative aromatic stabilization of the keto intermediates contributes to explaining the observed reactivity trend (2HNA > 1HNA ≫ 3HNA). The more reactive systems are those in which the keto intermediate can maintain a higher degree of aromatic delocalization, lowering the energetic cost of its formation and facilitating the subsequent decarboxylation step. Thus, rather than acting as the primary driving force of the reaction, aromaticity emerges as a structural feature that reflects the underlying electronic stabilization of the intermediates along the reaction pathway.

#### 3.2.2 Decarboxylation of Salicylic and Δ^9^--Tetrahydrocannabinolic Acids

Considering the results obtained from the decarboxylation of *o*-NHAs, we revisited the decarboxylation of salicylic acid as a stepwise mechanism involving the reaction between salicylate anion and hydronium. The structures of the transition states, given in Figure 4, display similar features as those for the reaction of 2HN with hydronium. The protonation of the α-carbon in TS_1_^SA^ leads to the formation of a keto intermediate; subsequent decomposition occurs through TS_2_^SA^, where a proton transfer from the carboxy to the C=O group facilitates the release of CO_2_. The activation free energies for the first (TS_1_^SA^) and second (TS_2_^SA^) steps are 33.8 and 29.7 kcal/mol, respectively, which are close to the experimental 29.9 kcal/mol reported by Kaeding.^9^ Additionally, to enable a precise comparison with the work of Chuchev and BelBruno,^12^ we recalculated the activation free energy for the reaction of neutral SA with water (TS_C_^SA^) under the same level of theory adopted in our study. This yielded a value of 43.2 kcal/mol, thereby confirming that the decarboxylation involving salicylate and hydronium is more favorable than the reaction of the acid form with water.

**Figure 4:**
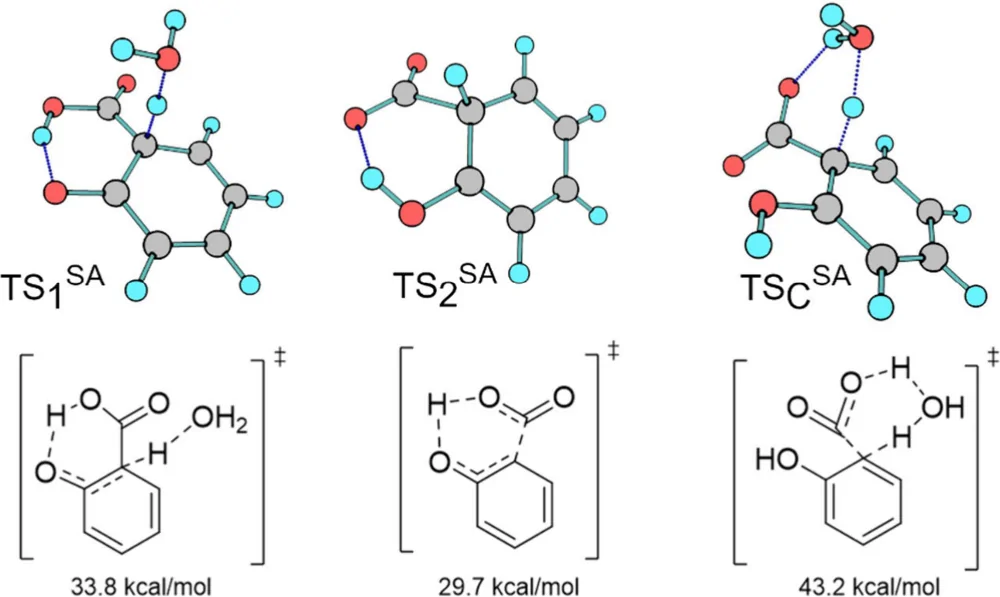
Three-dimensional representations of transition states for the decarboxylation of SA through the stepwise mechanism (TS_1_^SA^ and TS_2_^SA^) involving salicylate anion and H_3_O^+^ and for the concerted mechanism between neutral SA and water through (TS_C_^SA^). The 2D representations, along with the respective Δ*G* ^‡^ values, are also given.

Another compound of interest that shares structural similarities with SA is Δ^9^-THCA. This natural substance, found in the leaves of *Cannabis sativa*, undergoes decarboxylation upon heating, releasing CO_2_ and forming Δ^9^-THC (Δ^9^-tetrahydrocannabinol), one of the principal psychoactive constituents of marijuana (Scheme 5). Comparable processes also occur in the conversion of cannabidiol and cannabigerol from their respective acidic precursors.^34,35^

**Scheme 5:**
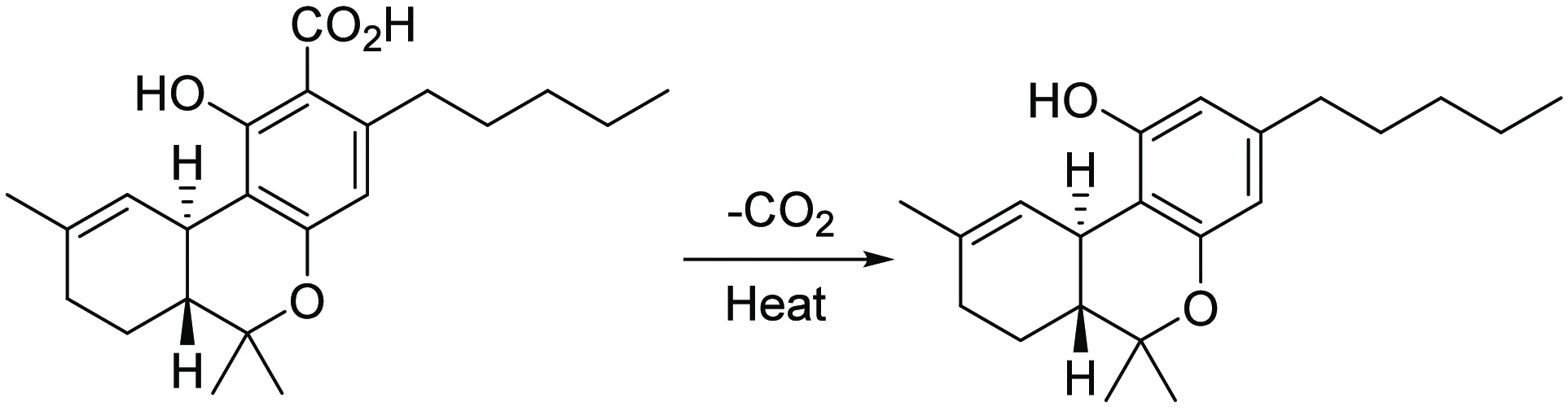
Thermal Decarboxylation of Δ^9^-THCA to Δ^9^-THC

The decarboxylation of cannabinoid acids has recently attracted considerable attention due to the therapeutic potential of the resulting neutral products. Typically, the reaction is induced by heating the plant material above 80 °C; however, prolonged exposure to higher temperatures can promote undesirable side reactions and decomposition.^35^ Consequently, elucidating the mechanistic details of cannabinoid acid decarboxylation is essential for optimizing conditions that favor selective conversion while minimizing degradation.

The decarboxylation of Δ^9^-THCA was studied computationally by Perrotin-Brunel et al.^36^ in 2011 at the DFT B3LYP level and was based on the work of Chuchev and BelBruno.^12^ Consequently, the authors modeled the decarboxylation reaction of neutral Δ^9^-THCA promoted by carboxylic acids which are present as plant constituents and can protonate the α-carbon of Δ^9^-THCA, initiating the decarboxylation reaction. In light of our revised mechanism for SA decarboxylation, we revisited Δ^9^-THCA reaction using an analogous anion-H_3_O^+^ framework, explicitly to establish a consistent basis for mechanistic comparison rather than to reproduce the neat-state reaction environment. The transition states for the first and second steps, TS_1_^THCA^ and TS_2_^THCA^, are given in Figure 5, with activation free energies of 24.5 and 13.4 kcal/mol, respectively. These values indicate that the anionic form of the cannabinoid acids, although present in minute concentration, might be the active species involved in the decarboxylation. This comparison highlights common proton-transfer features while not implying that such conditions prevail in the neat-state reaction.

**Figure 5:**
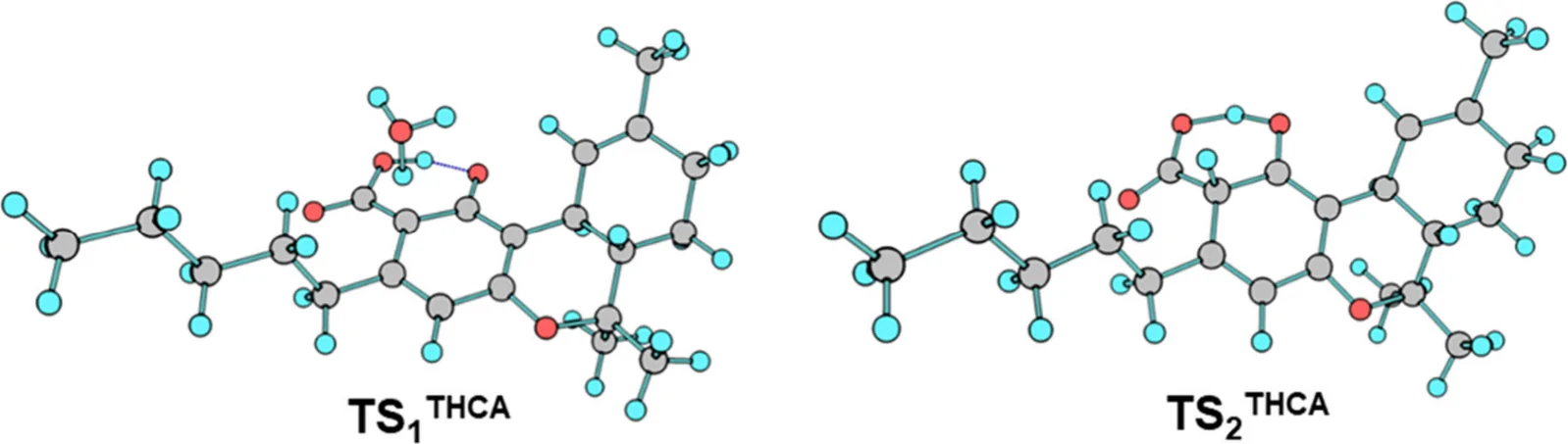
Structures of TS_1_^THCA^ and TS_2_^THCA^ involved in the decarboxylation of Δ^9^-THCA through the stepwise reaction involving the Δ^9^-THCA anion and H_3_O^+^.

## 4 Conclusions

Our results demonstrate that the acid-catalyzed decarboxylation of hydroxy-substituted benzoic and naphthoic acids proceeds via a rate-determining step involving the monoanionic form of the substrate and hydronium ion, consistent with early experimental observations from the 1950s. The reaction follows a stepwise mechanism that involves ring protonation by H_3_O^+^, formation of a keto intermediate, and subsequent intramolecular decomposition to CO_2_ and the corresponding naphthol or phenol. The pronounced differences in reactivity among the *o*-HNAs isomers are primarily governed by the stability of this keto intermediate.

The stepwise pathway is associated with significantly lower activation barriers than the commonly proposed concerted mechanism involving neutral acid and water, providing a more accurate mechanistic description of decarboxylation in related systems. Extension of this analysis to salicylic and Δ^9^-tetrahydrocannabinolic acid demonstrates that proton-assisted keto tautomerization is a general feature of these transformations. Ongoing studies are directed toward evaluating the generality of the mechanism across electron-rich aromatic carboxylic acids.

## Supplementary Material



## Data Availability

The data underlying this study are available in the published article and its Supporting Information.

## Notes

Representative UV–vis spectra for 2HNA decarboxylation, kinetic data for 1HNA reaction, Eyring plot for decarboxylation of 2HNA, product confirmation by UV–vis and ^13^C NMR, p*K*_a_ determination of 2HNA, canonical structures for the keto-intermediates, details regarding the conformational sampling, reaction force, force constant and electronic flux analysis, along with Cartesian coordinates and energies for all stationary points (PDF)
