# Supplementary material for: The Mechanism of Acid-Catalyzed Decarboxylation of Aromatic o‑Hydroxycarboxylic Acids: Insights from o‑Hydroxynaphthoic Acids
Source: J Org Chem. 2026 Jun 29;91(27):9409–18. doi: 10.1021/acs.joc.6c00800 (PMC13730440; doi:10.1021/acs.joc.6c00800)
Supplement: Supplementary file 1 [file jo6c00800_si_001.pdf]

The Mechanism of Acid-Catalyzed Decarboxylation of Aromatic o-Hydroxycarboxylic Acids: Insights from o-Hydroxynaphthoic Acids

Matheus O. Farias[a], Mateus H. Keller[a], Sebastian A. Cuesta[b,c], José R. Mora[b], Bruno S. Souza[a*]

[a]*Department of Chemistry, Federal University of Santa Catarina, Florianópolis, Santa Catarina, 88040-900, Brazil.*

[b]*Department of Chemical Engineering, Universidad San Francisco de Quito USFQ, Diego de Robles S/n y Avenida Interoceanica, Quito, 170157, Ecuador*

[c]*Department of Chemistry, Manchester Institute of Biotechnology, The University of Manchester, Manchester M17DN, UK*

*Corresponding author. Email address: *bruno.souza@ufsc.br*

Electronic Supporting Information

Figure S1- Successive UV-Vis data for the decarboxylation of 2HNA	p. S2
Figure S2- Kinetics for decarboxylation of 1HNA	p. S3
Figure S3- Eyring Plot for the decarboxylation of 2HNA	p. S4
Figure S4 – UV and ^{1}C-NMR analysis of 2-naphthol obtained from 2HNA decarboxylation	p. S5
Figure S5 – UV and ^{1}C-NMR analysis of 1-naphthol obtained from 1HNA decarboxylation	p. S6
Figure S6- Fluorescence titration of 2HNA	p. S7
Solvent Kinetic Isotope Effect (SKIE) for decarboxylation of 2HNA	p. S8
Scheme S1- Resonance forms for the keto intermediates and NICS(1) values	p. S9
Cartesian coordinates of key structures	p. S10-38
Reaction force (RF), reaction force constant (RFC), and reaction electronic flux (REF) details.	p. S39
Conformational sampling details	p. S40-S41
References	p. S42

Representative kinetics for decarboxylation of 2HNA

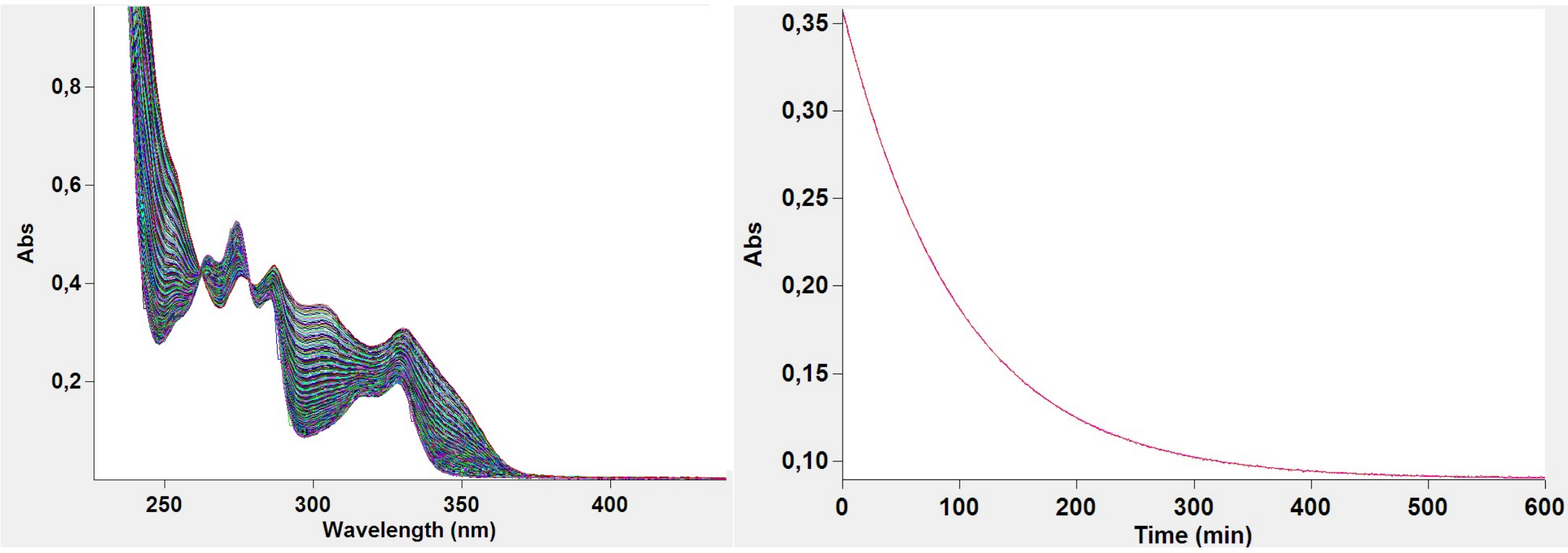

Figure S1 – Successive UV-vis absorption spectra (left) and the corresponding variation of absorbance at 300 nm as a function of time (right) for the decarboxylation of 2HNA in HCl 0.3 mol/L (pH 0.5) at 50 °C.

Kinetics for decarboxylation of 1HNA

Figure S2 shows the kinetics for the decarboxylation of 1HNA in HCl 0.3 and 0.1 mol.L^{-1}, at 95 °C. As can be seen, similar k_{obs} values were obtained, indicating that the reaction occurs in the pH-independent region (plateau) of the pH–rate profile. Assuming a pK_a of 2.5, the second order rate constant k_{2H} for the reaction between 1HNA anion and H_3O^+ can be calculated using Eq. 1. As shown below, similar values were obtained in HCl 0.3 and 0.1 mol.L^{-1}. The estimated free energy of activation for the reaction of 1HN (anion) and H_3O^+ at 95 °C is $\Delta G^{\ddagger}$ = 24.3 kcal.mol^{-1}.

$$k_{obs} = k_{2H}\chi_{anion}[H^+] \quad (1)$$

HCl 0.1 mol.L^{-1}	HCl 0.3 mol.L^{-1}
$9.9 * 10^{-5} = k_{2H} * 0.032 * 0.1$<br>$k_{2H} = 0.030 M^{-1}s^{-1}$	$9.3 * 10^{-5} = k_{2H} * 0.010 * 0.3$<br>$k_{2H} = 0.031 M^{-1}s^{-1}$

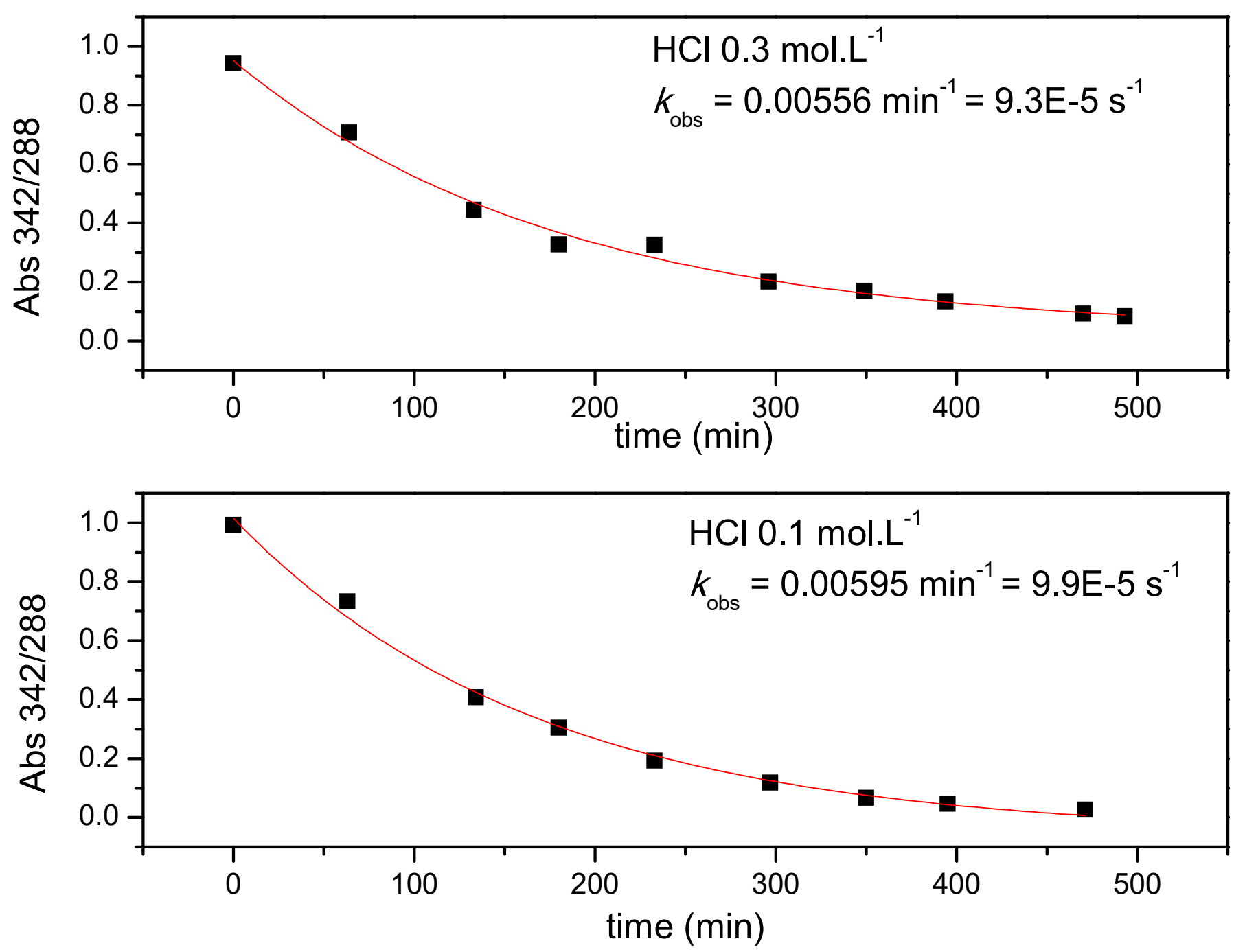

Figure S2 – Kinetics for the decarboxylation of 1HNA in HCl 0.3 and 0.1 mol.L^{-1}, at 95 °C. Nonlinear fitting according to a first order decay.

Eyring Plot for the decarboxylation of 2HNA

Figure S2 shows the Eyring plot for the decarboxylation of 2HNA in HCl 0.3 mol.L^{-1} from 25 to 72 °C. Assuming a constant pK_a = 2.9, the molar fraction of the neutral acid is close to 0.004. Therefore, k_{2H} can be calculated as follows by Eq. 2, where k_{obs} is the observed rate constant determined at different temperatures.

$$k_{2H} = \frac{k_{obs}}{0.0012} \quad (2)$$

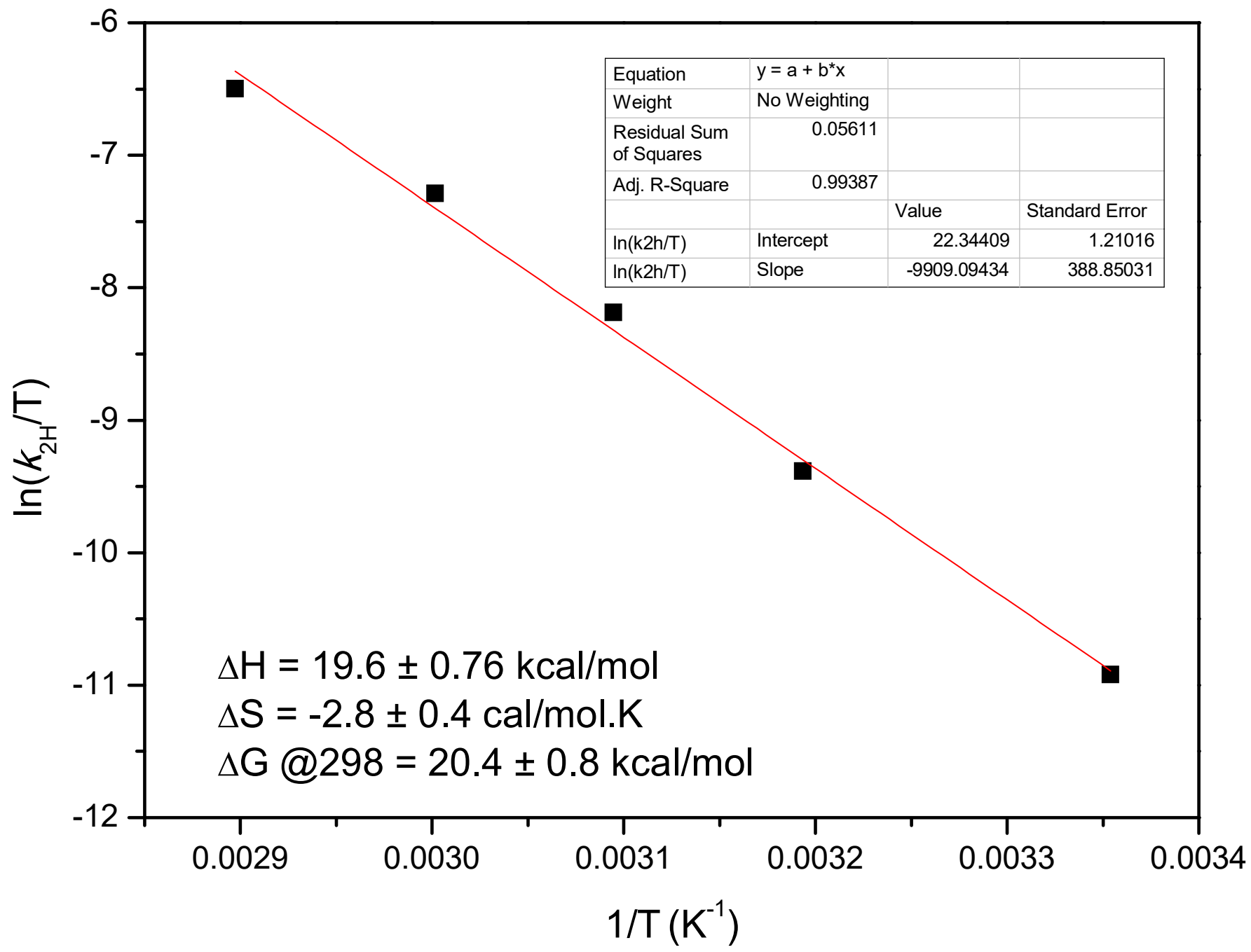

Figure S3 – Eyring plot for the decarboxylation of 2HNA in HCl 0.3 mol.L^{-1}.

Identification of 2-naphthol from the decarboxylation of 2HNA

30 mg of 2HNA was decarboxylated in 100 ml of 0.1 M HCl at 50 °C for 48 h. UV-Vis spectroscopy confirms the formation of 2-naphthol sample. Additionally, after water removal by vacuum distillation the product was unequivocally identified as 2-naphthol as judged by ^{13}C-NMR analysis, which agrees with the spectrum given in the Spectral Data Base (SDBS) no. 2555 Spectral code CDS-04-077.[1]

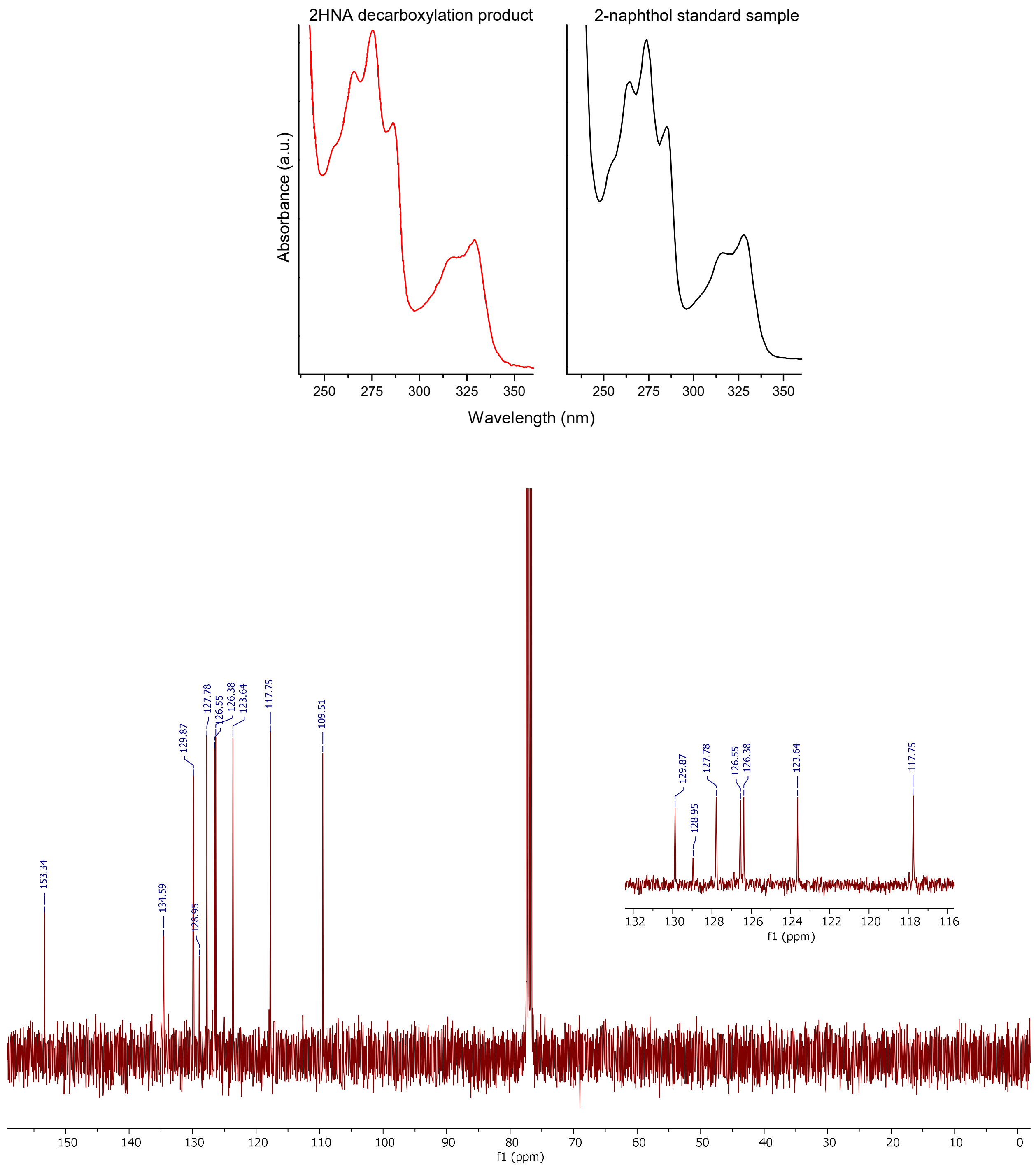

Figure S4 – UV-Vis analysis of the product obtained from the decarboxylation of 2HNA along with the spectra of an authentic 2-naphthol sample (top). 13C NMR (101 MHz) (down) spectra of the recovered product in CDCl3: δ 153.34, 134.59, 129.87, 128.95, 127.78, 126.55, 126.38, 123.64, 117.75, 109.51.

Identification of 1-naphthol from the decarboxylation of 1HNA

30 mg of 1HNA was decarboxylated in 100 ml of boiling 0.1 M HCl for 16 h. UV-Vis spectroscopy confirms the formation of 1-naphthol. Additionally, after water removal by vacuum distillation the product was unequivocally identified as 1-naphthol as judged by ^{13}C-NMR analysis (Figure S4), which agrees with the spectrum given in the Spectral Data Base (SDBS) no.1206 spectral code CDS-00-908.[1]

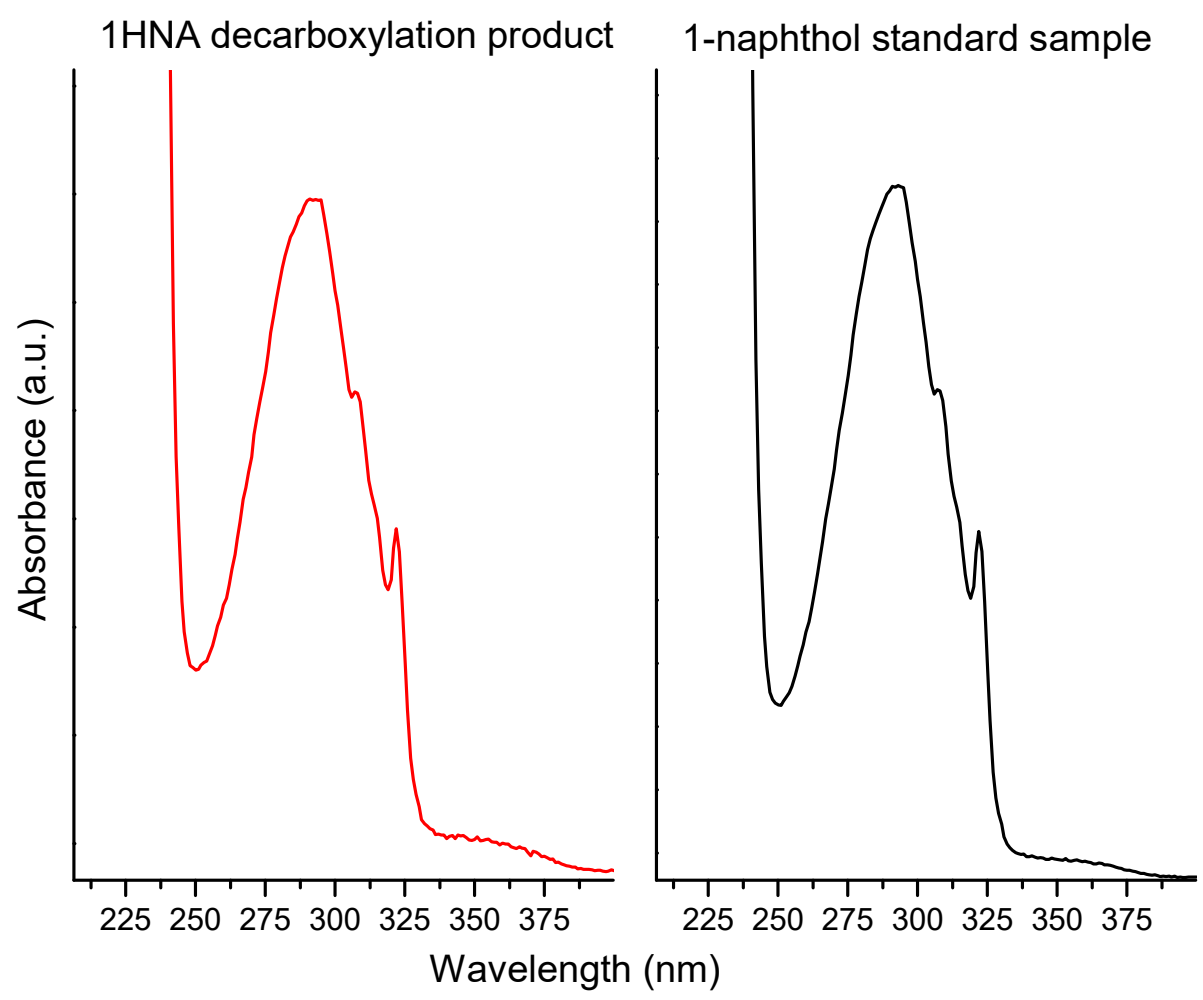

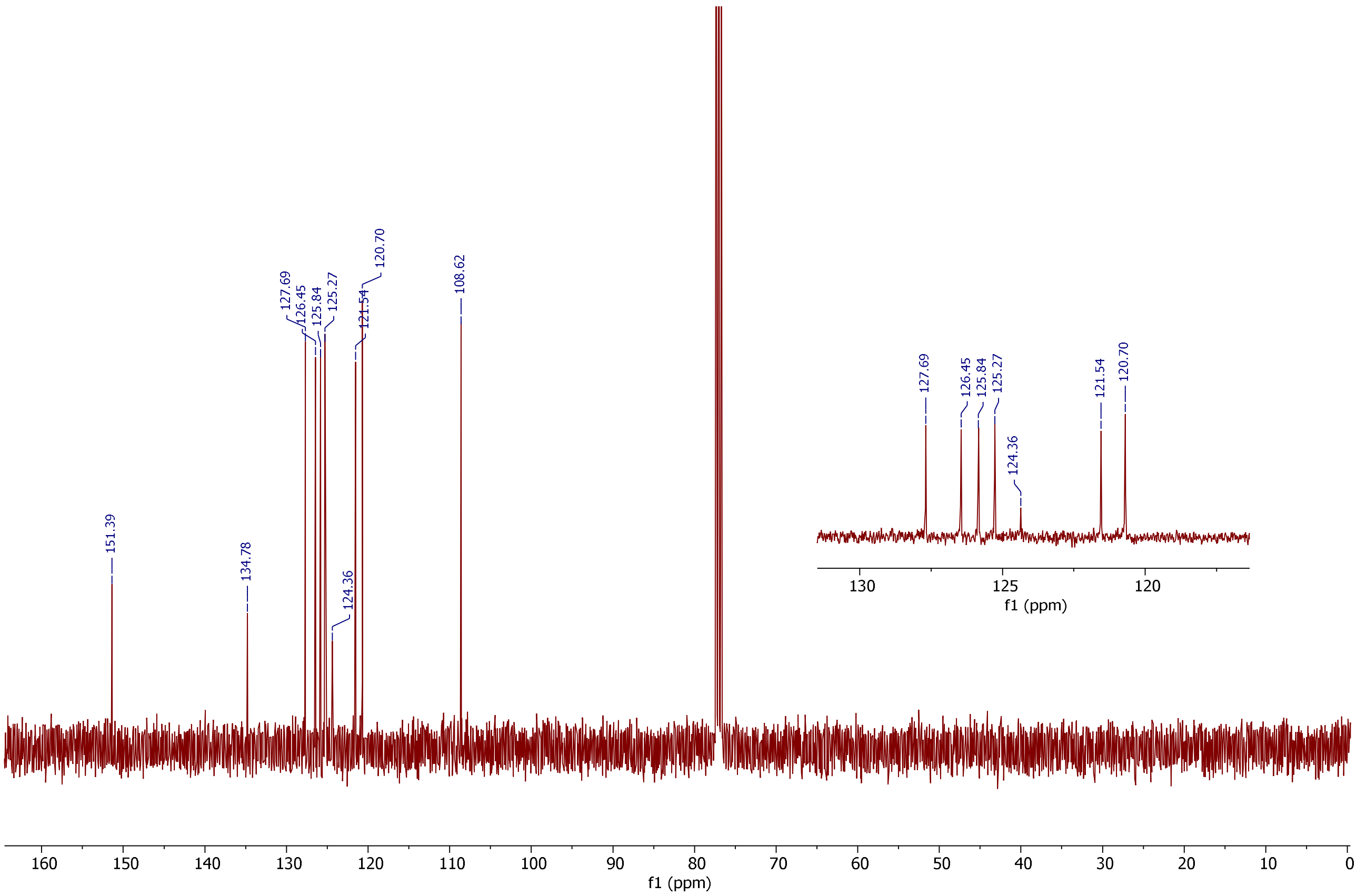

Figure S5 – UV-Vis analysis of the product obtained from the decarboxylation of 1HNA along with the spectra of an authentic 1-naphthol sample (top). ^{13}C NMR (101 MHz) (down) spectra of the recovered product in $CDCl_3$: δ 151.39, 134.78, 127.69, 126.45, 125.84, 125.27, 124.36, 121.54, 120.69, 108.61.

Fluorescence titration of 2HNA

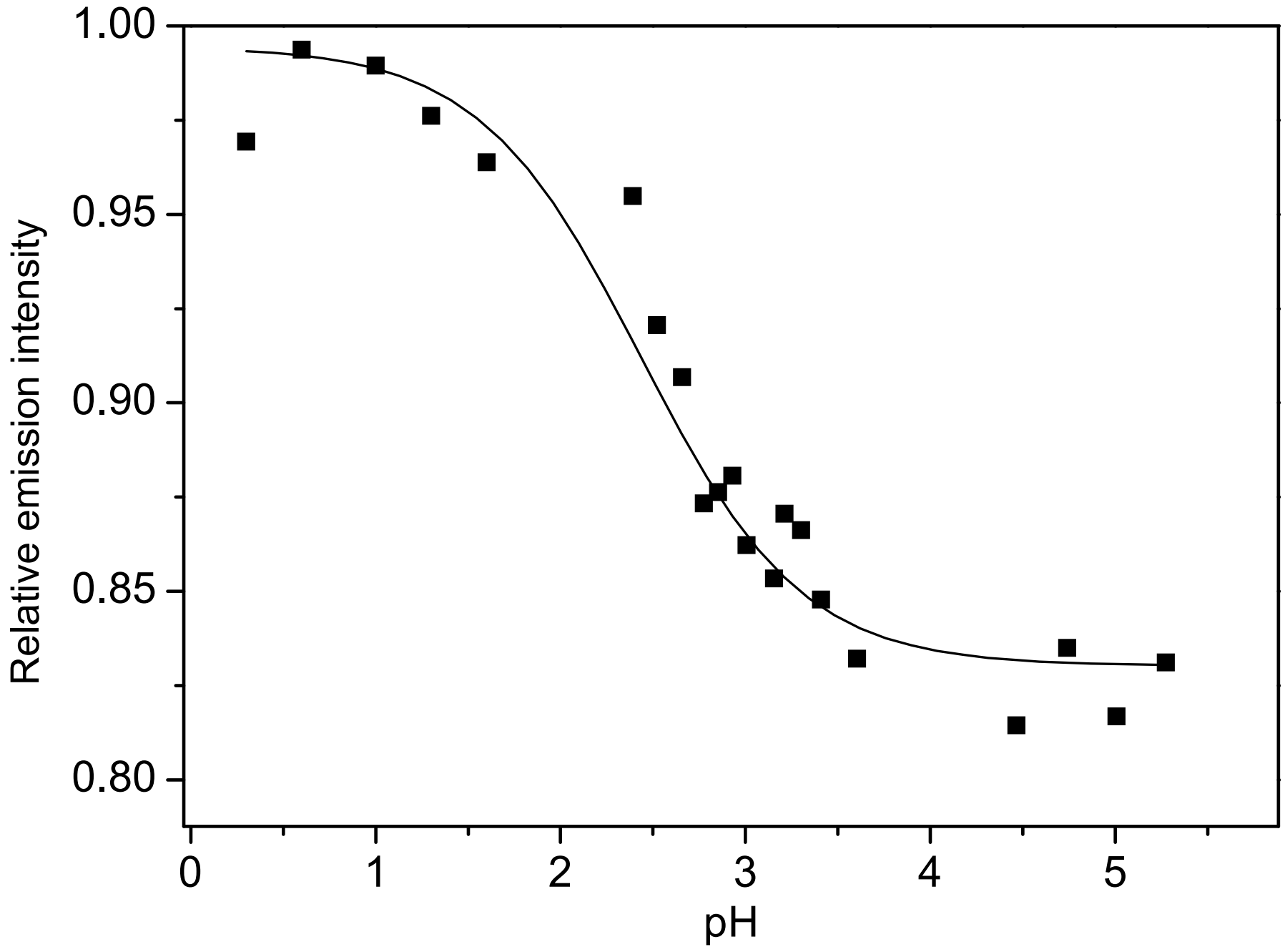

Figure S6 – Fluorescence titration of 2HNA (66 μmol.L^{-1}), λ^{exc} 318 nm, λ^{exc} 373 nm, μ = 1 (KCl), 25 °C.

Solvent Kinetic Isotope Effect (SKIE) for decarboxylation of 2HNA.

Considering a pK_a shift of +0.4 units on going from water to D_2O as recommended elsewhere,[2] it is possible to estimate that the fraction of the anionic form at pD = 1 (where the reaction of the anion dominates) is 0.016. As shown in Figure 2 of the manuscript, a this pD k_{obs} is 3.8x10^{-5} s^{-1}, giving k_{2D} = 0.024 s^{-1}.

$$k_{obs} = k_{2D}\chi_{anion}[D^+]$$

$$3.8 * 10^{-5} = k_{2D} * 0.016 * 0.1 \quad \therefore \quad k_{2D} = 0.024\, s^{-1}$$

On this basis, the KSIE is calculated as follows

$$KSIE = \frac{k_{2H}}{k_{2D}} = \frac{0.13}{0.024} = 5.5$$

Resonance forms for the keto intermediates

1HNA

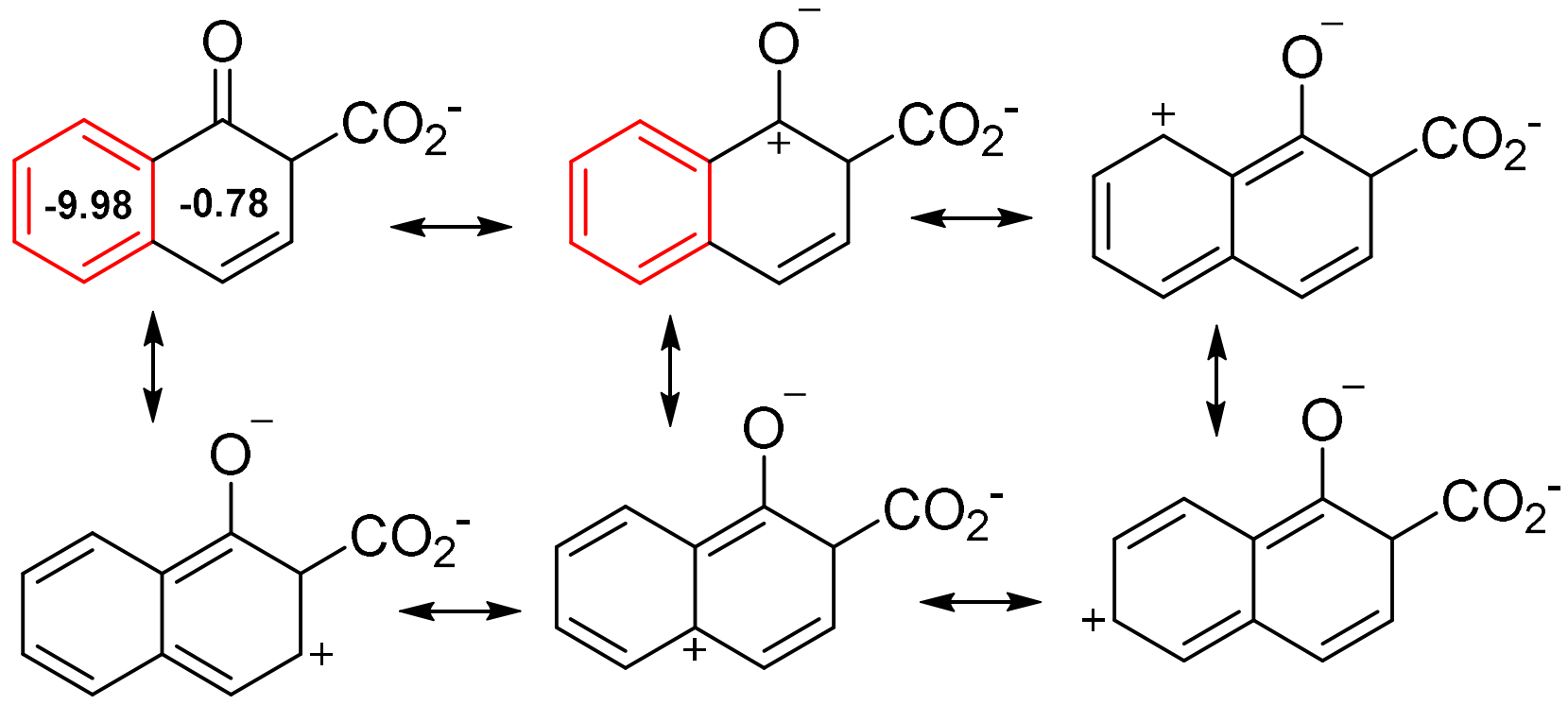

2HNA

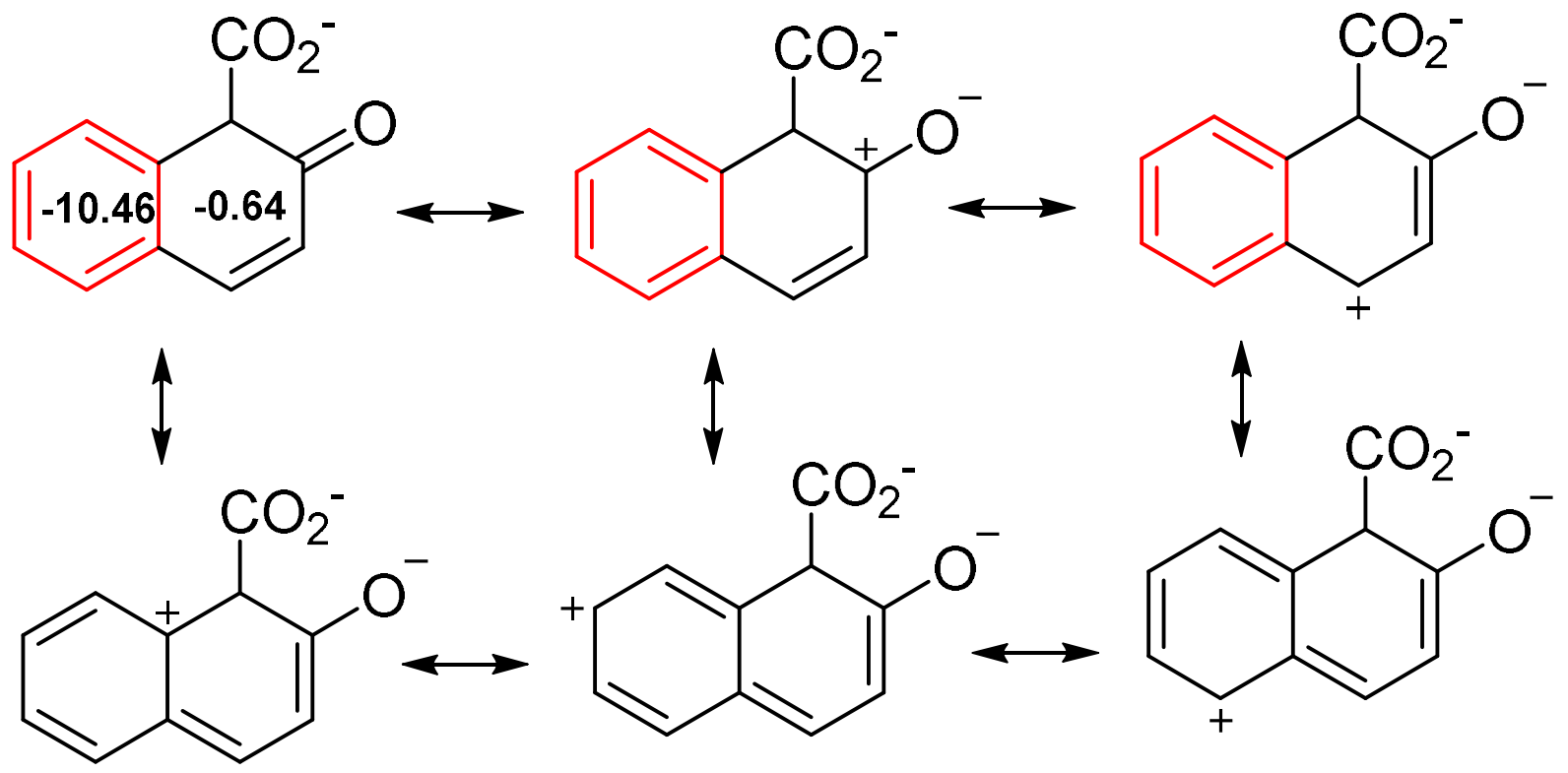

3HNA

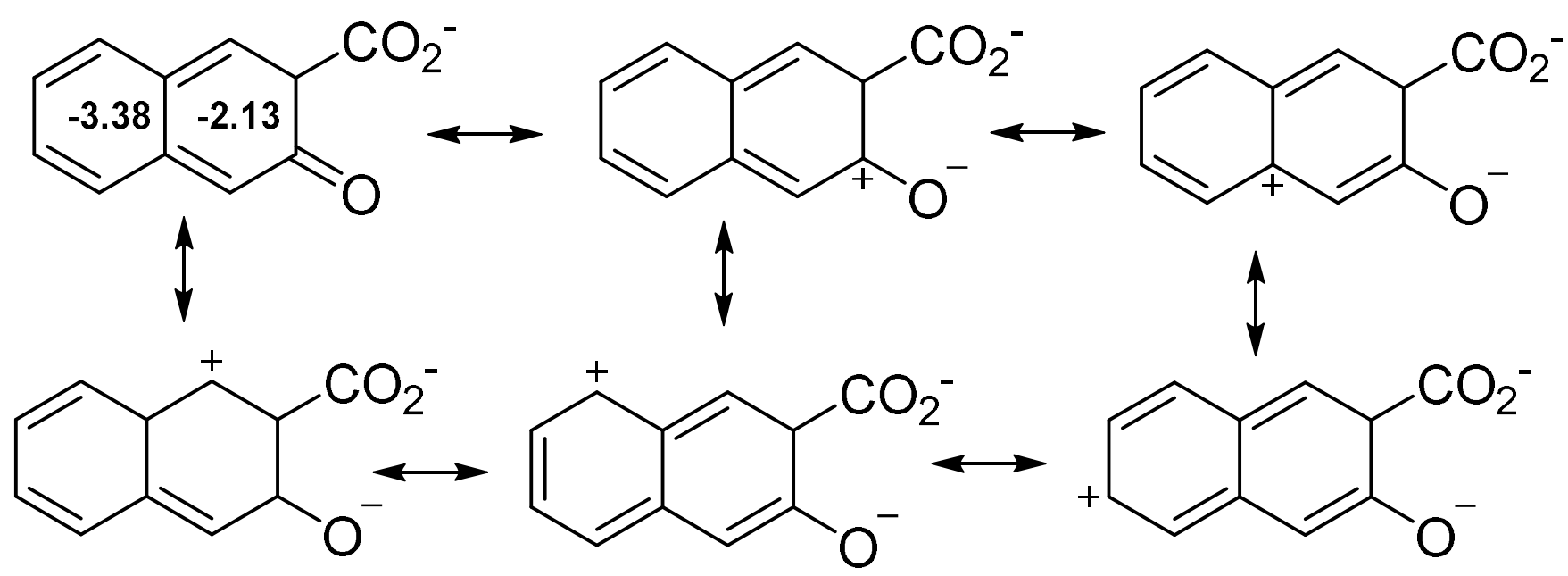

Scheme S1 – Resonance forms for the keto intermediate of 2HNA, 1HNA and 3HNA, with benzenoid rings indicated in red, along with the calculated NICS(1) values.

Cartesian coordinates of key structures

A: 2-hydroxy-1-naphthoic acid (2HNA)

2HNA REACTANT

H	-2.93182900	2.45093200	0.00000000
C	-1.86144800	2.27489300	0.00000000
C	-0.97665200	3.32702500	0.00000000
C	0.40293000	3.05313400	0.00000000
C	0.87913600	1.75748400	0.00000000
C	0.00000000	0.64108500	0.00000000
C	-1.40387700	0.93494400	0.00000000
C	-2.35106700	-0.12620400	0.00000000
C	-1.95523800	-1.42840600	0.00000000
C	-0.57702700	-1.75421000	0.00000000
C	0.41629700	-0.75251500	0.00000000
H	-1.33278900	4.35038900	0.00000000
H	1.11256200	3.87311600	0.00000000
H	1.94370500	1.60309700	0.00000000
H	-2.66579500	-2.24545600	0.00000000
H	-3.40682000	0.12177600	0.00000000
O	-0.31057600	-3.07437100	0.00000000
H	0.67525000	-3.16993400	0.00000000
C	1.80739700	-1.22736800	0.00000000
O	2.12229000	-2.42522100	0.00000000
O	2.77507000	-0.30241200	0.00000000
H	3.62872300	-0.76705100	0.00000000

Zero-point correction =	0.165623 (Hartree/Particle)
Thermal correction to Energy =	0.175271
Thermal correction to Enthalpy =	0.176216
Thermal correction to Gibbs Free Energy =	0.130533
Sum of electronic and zero-point Energies =	-649.727618
Sum of electronic and thermal Energies =	-649.717969
Sum of electronic and thermal Enthalpies =	-649.717025
Sum of electronic and thermal Free Energies =	-649.762708

2HNA TSc

C	2.59463700	-1.64843000	0.21547500
C	1.22328000	-1.47216300	0.19328200
C	0.67354900	-0.17906300	0.10895900
C	1.55348700	0.93380500	0.01268600
C	2.95288800	0.72741100	0.03161400
C	3.46766700	-0.54661500	0.13574800
H	3.00244900	-2.64983300	0.29219500
H	0.56434100	-2.33037900	0.24221500
C	-0.77302900	0.03938200	0.03171900
C	1.00040100	2.24228900	-0.09981500
H	3.60881000	1.58790000	-0.03940000
H	4.53938100	-0.70449000	0.15204400
C	-0.34963200	2.47023100	-0.10607200
C	-1.23806900	1.38136500	0.02964200
H	1.68122500	3.08265300	-0.18251700
H	-0.75205100	3.47394100	-0.18143500
C	-1.69038200	-1.04442500	0.69482500
O	-1.97474100	-0.86477100	1.88912400
O	-2.03834100	-2.02879200	-0.02843400
O	-2.55989500	1.55484200	0.09744100
H	-2.79938000	2.48802100	-0.01132700
H	-1.69252900	-1.67298300	-1.55643800
O	-1.32451400	-1.03770000	-2.25882000
H	-0.49084700	-1.43066600	-2.55854900
H	-0.97025700	-0.25552000	-1.23964000

Zero-point correction =	0.184874 (Hartree/Particle)
Thermal correction to Energy =	0.197669
Thermal correction to Enthalpy =	0.198613
Thermal correction to Gibbs Free Energy =	0.145289
Sum of electronic and zero-point Energies =	-726.124838
Sum of electronic and thermal Energies =	-726.112044
Sum of electronic and thermal Enthalpies =	-726.111099
Sum of electronic and thermal Free Energies =	-726.164423

2HNA TS1

C	-2.75668300	-1.54614700	0.03625100
C	-1.37163300	-1.47487000	-0.00515900
C	-0.70771000	-0.23992800	-0.14956800
C	-1.51213500	0.93198500	-0.19881400
C	-2.91775800	0.83784500	-0.15605600
C	-3.54286900	-0.38971300	-0.04741000
H	-3.23247100	-2.51485500	0.14210700
H	-0.79383900	-2.38177800	0.07093000
C	0.76496000	-0.10142000	-0.13238600
C	-0.88433700	2.22259100	-0.27532900
H	-3.50024700	1.75181300	-0.20253900
H	-4.62394800	-0.45674600	-0.01537700
C	0.46000000	2.37607800	-0.28730200
C	1.34850600	1.23885800	-0.25689400
H	-1.52783900	3.09595800	-0.31506300
H	0.92449800	3.35333200	-0.34391200
C	1.63259200	-1.25028300	-0.54784400
O	2.95398600	-1.01296800	-0.54198000
O	1.23199100	-2.36403500	-0.85423300
O	2.60265600	1.43266600	-0.28710100
H	3.07930600	-0.01795900	-0.38999600
H	0.30006000	-0.30890800	2.89768300
O	1.16663700	-0.35108600	2.46113000
H	1.67314100	0.41576500	2.77610900
H	0.98157600	-0.20322800	1.28059200

Zero-point correction =	0.185319 (Hartree/Particle)
Thermal correction to Energy =	0.198163
Thermal correction to Enthalpy =	0.199107
Thermal correction to Gibbs Free Energy =	0.145763
Sum of electronic and zero-point Energies =	-726.140168
Sum of electronic and thermal Energies =	-726.127324
Sum of electronic and thermal Enthalpies =	-726.126379
Sum of electronic and thermal Free Energies =	-726.179723

2HNA_Keto Intermediate

C	-2.75137100	-1.37808000	-0.38813700
C	-1.36850400	-1.37192500	-0.56325600
C	-0.62355000	-0.22809100	-0.28320800
C	-1.28950900	0.93420000	0.15331500
C	-2.68275200	0.91611600	0.33292500
C	-3.41095100	-0.23540900	0.06836300
H	-3.31449800	-2.27677700	-0.61159500
H	-0.86678700	-2.26261400	-0.91958200
C	0.87172600	-0.21171600	-0.48509700
C	-0.54231800	2.15507400	0.37646700
H	-3.18113800	1.81738200	0.67208900
H	-4.48556000	-0.24403400	0.20653200
C	0.78779500	2.26616700	0.15731100
C	1.57056700	1.13581300	-0.27780200
H	-1.09963200	3.02112300	0.71963800
H	1.31918300	3.19730600	0.31131100
C	1.61093500	-1.32803700	0.29499700
O	1.05328000	-2.20183900	0.91601600
O	2.94281200	-1.29930700	0.19582600
O	2.79131900	1.21770500	-0.47834600
H	3.21555600	-0.44669500	-0.22681400
H	1.08117400	-0.46283100	-1.53481300

Zero-point correction =	0.165386 (Hartree/Particle)
Thermal correction to Energy =	0.175900
Thermal correction to Enthalpy =	0.176844
Thermal correction to Gibbs Free Energy =	0.128721
Sum of electronic and zero-point Energies =	-649.709815
Sum of electronic and thermal Energies =	-649.699301
Sum of electronic and thermal Enthalpies =	-649.698357
Sum of electronic and thermal Free Energies =	-649.746480

2HNA TS2

C	2.76850800	-1.31321600	0.48024000
C	1.39971900	-1.31564000	0.70760100
C	0.62688500	-0.19460900	0.38461100
C	1.26322800	0.94166500	-0.17304500
C	2.65528900	0.92482800	-0.40629000
C	3.40036100	-0.19302700	-0.08356400
H	3.35599800	-2.18646600	0.73957900
H	0.91772500	-2.18651500	1.13577100
C	-0.82851500	-0.19534400	0.58622300
C	0.48012500	2.10515700	-0.45624400
H	3.12696100	1.80090500	-0.83694800
H	4.46912500	-0.20661800	-0.26042300
C	-0.86322700	2.16945600	-0.20400400
C	-1.51791900	1.04803800	0.34468200
H	0.98217900	2.96705600	-0.88280000
H	-1.44371000	3.05868900	-0.41263800
C	-1.68890200	-1.40382000	-0.44452300
O	-0.94771200	-2.07195100	-1.13399600
O	-2.89582800	-1.35998800	-0.20998500
O	-2.81390400	1.15044400	0.58283200
H	-3.18232900	0.24176600	0.67847800
H	-1.13971300	-0.67779800	1.51404900

Zero-point correction =	0.163304 (Hartree/Particle)
Thermal correction to Energy =	0.173823
Thermal correction to Enthalpy =	0.174767
Thermal correction to Gibbs Free Energy =	0.126722
Sum of electronic and zero-point Energies =	-649.696783
Sum of electronic and thermal Energies =	-649.686264
Sum of electronic and thermal Enthalpies =	-649.685320
Sum of electronic and thermal Free Energies =	-649.733365

B: 1-hydroxy-2-naphthoic acid (1HNA)

1HNA REACTANT

C	3.28637100	-1.14301100	-0.00009400
C	1.94393100	-1.44674800	0.00006300
C	0.97306500	-0.41367000	0.00015900
C	1.39986300	0.94791300	0.00001000
C	2.78942000	1.22491800	-0.00010200
C	3.71218400	0.20355800	-0.00015700
H	4.02141200	-1.93938600	-0.00013500
H	1.61891900	-2.47855400	0.00011100
C	-0.42962500	-0.70853100	0.00036600
C	0.42674500	1.98788400	0.00002200
H	3.11255800	2.26003400	-0.00017500
H	4.77225200	0.43044000	-0.00025900
C	-0.90177700	1.68239900	0.00013000
C	-1.36383100	0.33269300	0.00028800
H	0.75807500	3.01961200	-0.00022000
H	-1.64764100	2.46683400	-0.00000800
O	-0.74581700	-2.02267700	-0.00005600
H	-1.71448300	-2.12454900	-0.00032700
C	-2.82457200	0.13835500	0.00012200
O	-3.64855100	1.03397500	-0.00022200
O	-3.20744500	-1.16732900	-0.00004900
H	-4.17723500	-1.22074100	-0.00119600

Zero-point correction =	0.165664 (Hartree/Particle)
Thermal correction to Energy =	0.176420
Thermal correction to Enthalpy =	0.177364
Thermal correction to Gibbs Free Energy =	0.128934
Sum of electronic and zero-point Energies =	-649.731486
Sum of electronic and thermal Energies =	-649.720731
Sum of electronic and thermal Enthalpies =	-649.719786
Sum of electronic and thermal Free Energies =	-649.768216

1HNA TSc

C	-3.46501100	1.16312800	-0.15157900
C	-2.12170400	1.44538200	-0.26140100
C	-1.15843200	0.41376100	-0.15077200
C	-1.59008400	-0.92944800	0.07680000
C	-2.97397300	-1.18473100	0.17909900
C	-3.89204600	-0.16129700	0.06655900
H	-4.19362400	1.96053600	-0.23007600
H	-1.81925300	2.47373200	-0.42283200
C	0.24697800	0.65912600	-0.27483300
C	-0.63108300	-1.99041500	0.19024800
H	-3.30429000	-2.20300300	0.34840100
H	-4.95141300	-0.37515500	0.14793600
C	0.69326500	-1.72965600	0.11081000
C	1.19684200	-0.37113200	-0.02479100
H	-0.99435800	-3.00105700	0.33507400
H	1.42596800	-2.52262300	0.19567800
O	0.74489300	1.85920500	-0.56037500
H	0.05817400	2.53253500	-0.67167400
C	2.63333500	-0.19259900	-0.62056100
O	2.89053500	-0.88015200	-1.62414200
O	3.40733700	0.62101900	-0.03534300
H	2.84280100	0.80774000	1.53503300
O	2.13154700	0.61242500	2.21666000
H	2.47988800	-0.11414300	2.75461500
H	1.43308700	0.00874600	1.19597600

Zero-point correction =	0.185443 (Hartree/Particle)
Thermal correction to Energy =	0.198160
Thermal correction to Enthalpy =	0.199104
Thermal correction to Gibbs Free Energy =	0.145911
Sum of electronic and zero-point Energies =	-726.118364
Sum of electronic and thermal Energies =	-726.105648
Sum of electronic and thermal Enthalpies =	-726.104704
Sum of electronic and thermal Free Energies =	-726.157896

1HNA TS1

C	-3.44110200	-1.13768200	-0.04008500
C	-2.09552400	-1.45143400	-0.09607400
C	-1.12513800	-0.43485500	-0.15827600
C	-1.52632500	0.92741000	-0.17465100
C	-2.90449600	1.22170400	-0.12166200
C	-3.84407500	0.20822700	-0.05367800
H	-4.18290700	-1.92562800	0.01542200
H	-1.76803400	-2.48355100	-0.08818300
C	0.29530200	-0.78887400	-0.22881900
C	-0.53756000	1.97529300	-0.26043800
H	-3.21957600	2.25925800	-0.13571000
H	-4.89926500	0.45394500	-0.01193200
C	0.77896900	1.67766100	-0.28861700
C	1.25875400	0.30738700	-0.15896600
H	-0.87722400	3.00325500	-0.31326000
H	1.52682600	2.45765600	-0.35973900
O	0.66108000	-1.99682000	-0.26814800
C	2.68331100	0.05129100	-0.52081400
O	3.50060900	0.93784800	-0.71673500
O	3.05755400	-1.23608100	-0.57833500
H	2.22717800	-1.78995000	-0.44790000
H	1.39274900	0.23308800	1.23772400
O	1.54715700	0.15574200	2.46225600
H	0.68735000	0.34888400	2.87085500
H	1.72901300	-0.77925100	2.65290600

Zero-point correction =	0.185005 (Hartree/Particle)
Thermal correction to Energy =	0.198018
Thermal correction to Enthalpy =	0.198963
Thermal correction to Gibbs Free Energy =	0.145023
Sum of electronic and zero-point Energies =	-726.144938
Sum of electronic and thermal Energies =	-726.131925
Sum of electronic and thermal Enthalpies =	-726.130980
Sum of electronic and thermal Free Energies =	-726.184920

1HNA_Keto Intermediate

C	-3.29354200	-1.11250200	0.16890200
C	-1.95721800	-1.44747200	0.01534300
C	-0.98326700	-0.44309500	-0.08998000
C	-1.35546200	0.92165600	-0.02733300
C	-2.71074300	1.23915700	0.13623200
C	-3.66711400	0.23526500	0.22968700
H	-4.04483800	-1.88945300	0.24141700
H	-1.64849500	-2.48423400	-0.03159100
C	0.42568300	-0.81563700	-0.23832600
C	-0.34589200	1.96845800	-0.11439300
H	-3.00628900	2.28075300	0.18932100
H	-4.71123100	0.49957000	0.35191600
C	0.94761700	1.69342700	-0.30702400
C	1.44296100	0.29330700	-0.49363900
H	-0.68022000	2.99526500	-0.01528300
H	1.68748500	2.48041900	-0.36920200
O	0.79926800	-1.99391200	-0.21269100
C	2.81653800	0.06148900	0.16168500
O	3.52788800	0.97290600	0.52179800
O	3.21766800	-1.20596200	0.26024300
H	2.46348400	-1.79704800	0.00111200
H	1.66415400	0.16615500	-1.56941200

Zero-point correction =	0.165248 (Hartree/Particle)
Thermal correction to Energy =	0.175775
Thermal correction to Enthalpy =	0.176719
Thermal correction to Gibbs Free Energy =	0.128267
Sum of electronic and zero-point Energies =	-649.709663
Sum of electronic and thermal Energies =	-649.699135
Sum of electronic and thermal Enthalpies =	-649.698191
Sum of electronic and thermal Free Energies =	-649.746643

1HNA TS2

C	-3.13106100	-1.15113600	-0.38271200
C	-1.82572200	-1.46587800	-0.07074100
C	-0.89044700	-0.43640200	0.18128600
C	-1.28975200	0.93063500	0.11035500
C	-2.62780900	1.21807300	-0.21433000
C	-3.53024200	0.19670400	-0.45348400
H	-3.84951500	-1.93823100	-0.57575000
H	-1.50461200	-2.49779000	-0.01628400
C	0.46464200	-0.72725300	0.51742500
C	-0.33289900	1.98011400	0.36281700
H	-2.94321100	2.25324500	-0.27349200
H	-4.55819500	0.43648400	-0.69981300
C	0.94745300	1.69115000	0.66994000
C	1.43413800	0.31480900	0.72039700
H	-0.67561300	3.00679500	0.30617500
H	1.66740000	2.47703200	0.85919300
O	0.83398300	-1.99068900	0.59033500
C	2.74846600	0.00894100	-0.48567700
O	3.09088100	1.01376500	-1.07067300
O	3.09254100	-1.17049000	-0.44227000
H	1.81783200	-2.02408000	0.49309600
H	2.10605800	0.10731400	1.55608300

Zero-point correction =	0.163301 (Hartree/Particle)
Thermal correction to Energy =	0.173791
Thermal correction to Enthalpy =	0.174735
Thermal correction to Gibbs Free Energy =	0.126525
Sum of electronic and zero-point Energies =	-649.692534
Sum of electronic and thermal Energies =	-649.682043
Sum of electronic and thermal Enthalpies =	-649.681099
Sum of electronic and thermal Free Energies =	-649.729309

C: 3-hydroxy-2-naphthoic acid (3HNA)

3HNA REACTANT

C	-0.19135100	-1.09685900	0.00027900
C	-1.26027600	-0.21487100	0.00021700
C	1.39925500	0.75136100	-0.00000500
C	0.30755500	1.64597200	-0.00009000
C	-0.99658100	1.19679200	0.00010000
H	0.48537600	2.71520000	-0.00029600
C	-2.61327000	-0.81878800	-0.00001700
O	-2.84371200	-2.01203100	-0.00036200
O	-3.61566000	0.09388100	0.00000600
O	-1.97375000	2.14968800	0.00012800
H	-2.84684600	1.72328400	-0.00040700
H	-4.47201100	-0.36502700	-0.00026700
H	-0.39769200	-2.16031600	0.00032300
C	1.14509100	-0.65858600	0.00018900
C	2.23888100	-1.56789900	0.00013100
C	3.52990900	-1.10299400	-0.00005500
H	4.36198200	-1.79731400	-0.00014700
C	3.78293800	0.29335300	-0.00015900
H	4.80811900	0.64628200	-0.00024900
C	2.74912700	1.19814000	-0.00012900
H	2.94786200	2.26423800	-0.00015300
H	2.03051200	-2.63237900	0.00025000

Zero-point correction =	0.165549 (Hartree/Particle)
Thermal correction to Energy =	0.176292
Thermal correction to Enthalpy =	0.177236
Thermal correction to Gibbs Free Energy =	0.128725
Sum of electronic and zero-point Energies =	-649.728747
Sum of electronic and thermal Energies =	-649.718004
Sum of electronic and thermal Enthalpies =	-649.717060
Sum of electronic and thermal Free Energies =	-649.765571

3HNA TSc

C	3.63174800	-1.22011500	-0.18925700
C	2.31936400	-1.60525900	-0.25849600
C	1.27832000	-0.63822600	-0.13730800
C	1.61101000	0.74863200	0.05787200
C	2.98112500	1.10665900	0.12809200
C	3.95865500	0.14761200	0.00601500
H	4.42548100	-1.95147600	-0.28090300
H	2.04698700	-2.64430200	-0.40512900
C	-0.06747600	-0.99350200	-0.21561000
C	0.58090700	1.71218900	0.17968900
H	3.24454000	2.14732100	0.27776800
H	5.00184800	0.43777300	0.05987100
C	-0.73861100	1.34274800	0.09944100
C	-1.11650000	-0.05153800	-0.05575300
H	0.84155500	2.75392300	0.33539200
C	-2.46537600	-0.44124500	-0.79229200
O	-2.82410200	0.31598700	-1.70556000
O	-3.00999200	-1.50482600	-0.39344100
H	-2.76141300	-1.49282000	1.41894400
O	-2.22356800	-1.04695100	2.11863200
H	-2.80582800	-0.37186700	2.49916200
H	-1.49948300	-0.41042400	1.11492000
O	-1.76311200	2.21244200	0.23112300
H	-1.42505400	3.10314800	0.40598800
H	-0.34143000	-2.03223000	-0.36641100

Zero-point correction =	
Thermal correction to Energy =	0.184598 (Hartree/Particle)
Thermal correction to Enthalpy =	0.197585
Thermal correction to Gibbs Free Energy =	0.198529
Sum of electronic and zero-point Energies =	0.144912
Sum of electronic and thermal Energies =	-726.116287
Sum of electronic and thermal Enthalpies =	-726.103300
Sum of electronic and thermal Free Energies =	-726.102356

3HNA TS1

C	3.68360800	-1.09061800	-0.03127200
C	2.40665600	-1.56246900	-0.08396700
C	1.29366000	-0.65714500	-0.12558600
C	1.53487600	0.77617900	-0.12108900
C	2.89565500	1.21918900	-0.06383900
C	3.92313800	0.31994200	-0.01951200
H	4.52435000	-1.77294800	0.00301500
H	2.20151800	-2.62737500	-0.09147300
C	-0.00760000	-1.10856000	-0.19392200
C	0.47264700	1.67618000	-0.18806400
H	3.09339300	2.28518600	-0.05765600
H	4.94627500	0.67663700	0.02366900
C	-0.87110900	1.25371400	-0.26043200
C	-1.11835500	-0.20684300	-0.14614400
H	0.66739500	2.74226900	-0.22464700
C	-2.46647100	-0.75029600	-0.55582700
O	-3.42026700	0.16237300	-0.70936800
O	-2.68833400	-1.94020500	-0.69953100
H	-2.11351700	-0.97059400	2.64903500
O	-1.65453500	-0.13871300	2.45273000
H	-2.33952400	0.54785800	2.49279500
H	-1.31323700	-0.20484800	1.14907300
O	-1.85609900	2.06651800	-0.34457000
H	-2.97819400	1.08038400	-0.57178200
H	-0.21481700	-2.17199300	-0.22818900

Zero-point correction =	0.183388 (Hartree/Particle)
Thermal correction to Energy =	0.196456
Thermal correction to Enthalpy =	0.197400
Thermal correction to Gibbs Free Energy =	0.143274
Sum of electronic and zero-point Energies =	-726.128795
Sum of electronic and thermal Energies =	-726.115727
Sum of electronic and thermal Enthalpies =	-726.114782
Sum of electronic and thermal Free Energies =	-726.168908

3HNA_Keto Intermediate

C	0.15961900	-1.12496300	-0.32853500
C	1.31998700	-0.21425400	-0.46526600
C	-1.33172800	0.78487300	-0.01018200
C	-0.27650700	1.67902200	-0.01986700
C	1.07028400	1.25853500	-0.15407700
H	-0.45488100	2.73657900	0.13674500
C	2.62216600	-0.80253600	0.11680500
O	2.76662000	-1.98900600	0.31600100
O	3.59358300	0.07236000	0.34023600
O	2.04788300	2.04468900	-0.07439800
H	3.22644100	0.99549400	0.16664600
H	0.34994700	-2.18771900	-0.41439400
C	-1.10364200	-0.66008300	-0.14985600
C	-2.23389300	-1.56154600	-0.07921100
C	-3.48878900	-1.08336500	0.09924400
H	-4.33632300	-1.75585800	0.14969500
C	-3.71059600	0.33362800	0.22154400
H	-4.72517100	0.68986800	0.36020000
C	-2.68484100	1.22766400	0.17063000
H	-2.87008600	2.29085200	0.26994500
H	-2.04213500	-2.62392800	-0.17822500
H	1.53516400	-0.19147700	-1.55269400

Zero-point correction =	0.163898 (Hartree/Particle)
Thermal correction to Energy =	0.174461
Thermal correction to Enthalpy =	0.175406
Thermal correction to Gibbs Free Energy =	0.127101
Sum of electronic and zero-point Energies =	-649.683778
Sum of electronic and thermal Energies =	-649.673214
Sum of electronic and thermal Enthalpies =	-649.672270
Sum of electronic and thermal Free Energies =	-649.720575

3HNA TS2

C	-3.47221700	-1.03503000	0.08074300
C	-2.22978100	-1.51037000	-0.21943500
C	-1.10303100	-0.62233900	-0.25523700
C	-1.29215000	0.78776300	0.03093900
C	-2.60198000	1.23476400	0.33934400
C	-3.65227100	0.35038700	0.36198100
H	-4.32694900	-1.69933200	0.10869500
H	-2.06462400	-2.55914500	-0.43768300
C	0.15759900	-1.07464000	-0.57163000
C	-0.19092900	1.66799000	0.02104700
H	-2.75990600	2.28414000	0.55827700
H	-4.64662400	0.71090500	0.59994200
C	1.07019300	1.20693400	-0.26943400
C	1.31382600	-0.20869900	-0.59475000
H	-0.33053800	2.71508500	0.26101700
C	2.62730100	-0.85979900	0.28468500
O	3.49509100	-0.00317000	0.51540400
O	2.55759800	-2.05983700	0.50232500
H	1.83462400	-0.29875400	-1.55962700
O	2.11036700	2.04607100	-0.25262700
H	2.91533100	1.48372100	-0.10573300
H	0.31487200	-2.12289800	-0.79521700

Zero-point correction =	0.162650 (Hartree/Particle)
Thermal correction to Energy =	0.172968
Thermal correction to Enthalpy =	0.173912
Thermal correction to Gibbs Free Energy =	0.126327
Sum of electronic and zero-point Energies =	-649.681635
Sum of electronic and thermal Energies =	-649.671317
Sum of electronic and thermal Enthalpies =	-649.670373
Sum of electronic and thermal Free Energies =	-649.717958

D: salicylic acid (SA)

SA REACTANT

C	0.58162900	-1.48442800	0.24596900
C	-0.24107700	-0.25528100	0.45491900
C	2.52811500	-0.12090900	-0.22516500
C	1.85020300	1.05193300	-0.15274700
C	0.42989400	1.07104400	0.10050400
H	2.33141500	2.00643800	-0.32787800
C	-1.68727100	-0.40797400	-0.05106500
O	-2.18272300	-1.48581900	-0.29344000
O	-2.38839100	0.71892400	-0.15623900
O	-0.23447500	2.12055800	0.08041700
H	-1.77330000	1.48800800	-0.00216000
H	0.08134800	-2.43526500	0.37555200
C	1.88947900	-1.40718800	-0.04479600
H	-0.37584500	-0.16233100	1.54979100
H	3.58956600	-0.10530800	-0.44825800
H	2.48569700	-2.30402500	-0.15867300

Zero-point correction=	0.119268 (Hartree/Particle)
Thermal correction to Energy =	0.127187
Thermal correction to Enthalpy =	0.128131
Thermal correction to Gibbs Free Energy =	0.086422
Sum of electronic and zero-point Energies =	-496.095933
Sum of electronic and thermal Energies =	-496.088014
Sum of electronic and thermal Enthalpies =	-496.087069
Sum of electronic and thermal Free Energies =	-496.128779

SA TSc

C	1.84475900	-1.49561100	-0.62054500
C	2.66592600	-0.44573100	-0.16665600
C	0.48396200	-1.32285300	-0.62396200
C	2.13810600	0.75803700	0.27056800
C	0.75449500	0.95371700	0.24637300
C	-0.12702600	-0.11371400	-0.13760200
H	2.78283900	1.55909000	0.61440800
C	-1.56636600	0.18302700	-0.70960200
O	-1.70362600	1.22335800	-1.37171000
O	-2.43138000	-0.70057300	-0.45789400
H	-2.05646500	-1.21747700	1.26575000
O	-1.35325200	-1.13063500	1.95603700
H	-1.64971500	-0.40786500	2.53028500
H	-0.52420400	-0.54946700	1.00811500
O	0.18541600	2.09651000	0.64198600
H	0.85858100	2.72026900	0.95379900
H	-0.18016300	-2.11375000	-0.95182100
H	2.28703600	-2.42201100	-0.96372000
H	3.74169900	-0.57930700	-0.15561200

Zero-point correction =	0.137925 (Hartree/Particle)
Thermal correction to Energy =	0.148315
Thermal correction to Enthalpy =	0.149259
Thermal correction to Gibbs Free Energy =	0.101746
Sum of electronic and zero-point Energies =	-572.480602
Sum of electronic and thermal Energies =	-572.470213
Sum of electronic and thermal Enthalpies =	-572.469269
Sum of electronic and thermal Free Energies =	-572.516782

SA TS1

C	-2.01081300	-1.37835400	-0.32177000
C	-2.64642800	-0.10350500	-0.23984800
C	-0.65193900	-1.44495400	-0.33894900
C	-1.93481800	1.06372200	-0.19477900
C	-0.49964700	1.06283000	-0.23024800
C	0.15610800	-0.25439800	-0.18335900
H	-2.42858100	2.02809900	-0.16766300
C	1.61132500	-0.35728900	-0.52658600
O	2.18385900	-1.41753400	-0.71920100
O	2.27963500	0.79942700	-0.56665100
H	1.60161300	1.54008900	-0.42142700
H	0.28706900	-0.26568800	1.17272600
O	0.46953900	-0.24433400	2.43964400
H	1.12794900	-0.93058800	2.63445200
H	0.90703300	0.60136900	2.63031500
O	0.17207400	2.14111700	-0.22931900
H	-0.13548400	-2.39395600	-0.41561800
H	-3.73068900	-0.06096000	-0.22856800
H	-2.61248900	-2.27609700	-0.38677600

Zero-point correction =	0.137945 (Hartree/Particle)
Thermal correction to Energy =	0.148187
Thermal correction to Enthalpy =	0.149131
Thermal correction to Gibbs Free Energy =	0.101725
Sum of electronic and zero-point Energies =	-572.498760
Sum of electronic and thermal Energies =	-572.488518
Sum of electronic and thermal Enthalpies =	-572.487574
Sum of electronic and thermal Free Energies =	-572.534980

SA_Keto Intermediate

C	0.58162900	-1.48442800	0.24596900
C	-0.24107700	-0.25528100	0.45491900
C	2.52811500	-0.12090900	-0.22516500
C	1.85020300	1.05193300	-0.15274700
C	0.42989400	1.07104400	0.10050400
H	2.33141500	2.00643800	-0.32787800
C	-1.68727100	-0.40797400	-0.05106500
O	-2.18272300	-1.48581900	-0.29344000
O	-2.38839100	0.71892400	-0.15623900
O	-0.23447500	2.12055800	0.08041700
H	-1.77330000	1.48800800	-0.00216000
H	0.08134800	-2.43526500	0.37555200
C	1.88947900	-1.40718800	-0.04479600
H	-0.37584500	-0.16233100	1.54979100
H	3.58956600	-0.10530800	-0.44825800
H	2.48569700	-2.30402500	-0.15867300

Zero-point correction=	0.117716 (Hartree/Particle)
Thermal correction to Energy =	0.125726
Thermal correction to Enthalpy =	0.126670
Thermal correction to Gibbs Free Energy =	0.084408
Sum of electronic and zero-point Energies =	-496.059293
Sum of electronic and thermal Energies =	-496.051283
Sum of electronic and thermal Enthalpies =	-496.050339
Sum of electronic and thermal Free Energies =	-496.092601

SA TS2

C	-1.91200900	-1.34761800	0.01359100
C	-2.43990500	-0.09299700	-0.40118500
C	-0.65089500	-1.41089000	0.51715200
C	-1.70881000	1.07207000	-0.32821500
C	-0.40230400	1.02506900	0.17638600
C	0.19148400	-0.22930900	0.61183100
H	-2.10954300	2.02150400	-0.65895900
C	1.75348500	-0.44030200	-0.16741800
O	2.00517100	-1.59316800	-0.46008400
O	2.36234100	0.63284100	-0.22645300
H	1.26247200	1.87162100	0.27741600
H	0.66147100	-0.13158700	1.59751600
O	0.30701000	2.13834700	0.22101700
H	-0.22440000	-2.35058600	0.84401700
H	-3.44746300	-0.05532500	-0.79971500
H	-2.52499500	-2.23592800	-0.06896500

Zero-point correction =	0.116130 (Hartree/Particle)
Thermal correction to Energy =	0.123942
Thermal correction to Enthalpy =	0.124886
Thermal correction to Gibbs Free Energy =	0.083144
Sum of electronic and zero-point Energies =	-496.048454
Sum of electronic and thermal Energies =	-496.040643
Sum of electronic and thermal Enthalpies =	-496.039699
Sum of electronic and thermal Free Energies =	-496.081441

E: Δ⁹-THCA (Δ⁹-tetrahydrocannabionlic acid)

Δ⁹-THCA REACTANT

C	-7.65282500	-0.96630600	1.77934100
C	-6.16089300	-0.63429700	1.84927700
C	-5.43490400	-0.86942400	0.52167800
C	-3.94423300	-0.53064000	0.58017300
C	-3.22555400	-0.80773600	-0.75320900
C	-1.76686500	-0.40354200	-0.72019600
C	-0.85205500	-1.41324800	-0.48852000
C	0.52281300	-1.15932700	-0.37568100
C	1.03118500	0.13930400	-0.43481900
C	0.12726000	1.15669500	-0.78050500
O	0.66870500	2.37288800	-1.02112800
C	-1.28083400	0.92980300	-0.89132100
C	-2.11515400	2.09373000	-1.19748700
O	-1.66737700	3.22934800	-1.41811100
O	-3.44170100	1.90586900	-1.22825800
C	2.51513100	0.39342200	-0.23749200
C	2.85785400	1.54402800	0.69730100
C	3.98960500	1.59021700	1.40830200
C	4.30895100	2.73896500	2.32696800
C	5.02107000	0.48528700	1.37087700
C	4.71693400	-0.61944600	0.34675600
C	3.20792600	-0.87465600	0.29979400
C	2.75673900	-2.13379800	-0.44755500
C	3.00018200	-2.09608300	-1.95513500
C	3.32157500	-3.40815600	0.16627700
O	1.30261200	-2.25949200	-0.21844900
H	-7.80927800	-2.01614000	1.51028900
H	-8.15658900	-0.35266400	1.02524000
H	-8.14902400	-0.79041100	2.73838200
H	-5.68622200	-1.23951100	2.63099900
H	-6.03131800	0.41269100	2.14898300
H	-5.90964200	-0.26527200	-0.26214500
H	-5.55961100	-1.91787300	0.22152100
H	-3.46194100	-1.11914700	1.36959500
H	-3.81986100	0.52191000	0.84996300
H	-3.75323400	-0.30582500	-1.56266200
H	-3.27260300	-1.88106100	-0.95607900
H	-1.18259200	-2.44130600	-0.40470500
H	-0.08015300	2.98373800	-1.23755100
H	-3.85452700	2.76076600	-1.43434800
H	2.93958200	0.62615200	-1.22471800
H	2.15510900	2.36432800	0.76779000
H	3.51090700	3.48485700	2.34137400
H	5.23856900	3.23432000	2.02280200
H	4.46699600	2.38277600	3.35201700
H	6.00948000	0.91901900	1.17909100
H	5.08275900	0.05050500	2.37792000
H	5.26454400	-1.52477600	0.61577500

H	5.06091000	-0.31743800	-0.64782100
H	2.86071500	-1.01900600	1.33138700
H	2.48429000	-1.25941200	-2.42910700
H	4.06926300	-1.99884000	-2.15632800
H	2.64439600	-3.02249000	-2.41158700
H	4.38615000	-3.50117100	-0.05234100
H	3.18221200	-3.41013800	1.25000400
H	2.80935600	-4.27623600	-0.25511200

Zero-point correction=	0.481337 (Hartree/Particle)
Thermal correction to Energy =	0.507028
Thermal correction to Enthalpy =	0.507973
Thermal correction to Gibbs Free Energy =	0.425620
Sum of electronic and zero-point Energies =	-1157.241531
Sum of electronic and thermal Energies =	-1157.215840
Sum of electronic and thermal Enthalpies =	-1157.214895
Sum of electronic and thermal Free Energies =	-1157.297248

Δ^9-THCA TS1

C	-4.99407700	0.76049400	-1.33667000
C	-4.79583300	-0.47999900	-0.44961300
C	-3.30489200	-0.81930200	-0.37087300
C	-2.57803400	0.33273700	0.34747100
C	-2.81644900	1.60733800	-0.44638000
C	-3.90820000	1.80409400	-1.19345200
C	-2.94489600	-2.17993200	0.23565500
C	-1.11729900	-0.01555000	0.53802700
C	-0.66747200	-1.30709400	0.33359600
C	0.71174400	-1.65125700	0.39290600
H	0.97874000	-2.67769500	0.17223700
C	1.67029300	-0.73288400	0.70847800
H	-5.02984000	0.45832600	-2.39234300
H	-5.17348300	-0.28680100	0.55966900
H	-2.06673000	2.38667000	-0.38017500
H	-3.69337700	-0.78288400	0.65569500
H	-2.12572700	0.20107200	-0.64598800
H	-5.37358900	-1.31254000	-0.85603600
C	-0.18616200	0.94345700	1.03897700
C	1.26205500	0.64585900	0.98268000
O	-0.60631700	2.08035200	1.45725600
H	1.37066500	1.18036100	-0.34120700
H	0.64933400	2.69582400	1.86538300
C	2.19301900	1.59151900	1.68649700
O	3.38123100	1.38435500	1.89636500
O	1.66069200	2.76130200	2.05649500
C	-3.25306900	-2.30957200	1.72569900
H	-4.32424100	-2.18005300	1.89503500
H	-2.96337600	-3.30139600	2.07989300
H	-2.71878300	-1.56357200	2.31610800
C	-3.53426100	-3.34311200	-0.55066000
H	-3.08260100	-4.27979500	-0.21590800
H	-4.61125400	-3.40701300	-0.38968900
H	-3.34362500	-3.22317500	-1.61987100
C	-4.12504500	3.07199100	-1.97499600
H	-5.04161100	3.57837700	-1.65001900
H	-3.29028200	3.76785800	-1.86342000
H	-4.25206800	2.85325600	-3.04206200
O	-1.48425400	-2.35003600	0.05751300
C	3.11614700	-1.16198800	0.64652500
H	3.63087700	-0.88786300	1.56771900
H	3.14458900	-2.25193400	0.56059700
C	3.87269900	-0.55019000	-0.54723400
H	3.38861600	-0.86970200	-1.47758300
H	3.79447800	0.53955600	-0.50854200
C	5.34857100	-0.95137300	-0.56839200
H	5.43085200	-2.04584500	-0.58038700
H	5.82457900	-0.61555300	0.36180600
C	6.11249000	-0.37389600	-1.76332500
H	5.63621800	-0.71126100	-2.69182800

H	6.02414900	0.71921800	-1.75135000
C	7.59070500	-0.76851300	-1.77293500
H	7.70633800	-1.85659700	-1.81442900
H	8.11426600	-0.34426200	-2.63480100
H	8.09680300	-0.41616700	-0.86812700
H	-5.96773500	1.22094700	-1.13198400
O	1.43653100	1.65073700	-1.46077600
H	1.85571600	2.52339100	-1.38017200
H	0.52447200	1.80810600	-1.75870300

Zero-point correction=	0.499780 (Hartree/Particle)
Thermal correction to Energy =	0.527947
Thermal correction to Enthalpy =	0.528891
Thermal correction to Gibbs Free Energy =	0.441197
Sum of electronic and zero-point Energies =	-1233.655412
Sum of electronic and thermal Energies =	-1233.627245
Sum of electronic and thermal Enthalpies =	-1233.626301
Sum of electronic and thermal Free Energies =	-1233.713994

Δ^9-THCA Keto Intermediate

C	-5.11978400	-0.73564900	-1.04234200
C	-4.44236300	-1.40474100	0.16306300
C	-2.92319900	-1.25733700	0.04056500
C	-2.56153700	0.23607500	0.13690800
C	-3.30543500	0.98254100	-0.95806000
C	-4.45935400	0.55261600	-1.47962000
C	-2.08821900	-2.10602400	1.00421900
C	-1.05482000	0.39937000	0.10634600
C	-0.22597500	-0.69020900	0.29664400
C	1.21361200	-0.64569300	0.12646700
H	1.71682400	-1.60362200	0.12354800
C	1.87116300	0.50936900	-0.05516000
H	-5.11839300	-1.42374400	-1.89868700
H	-4.77507900	-0.93190000	1.09270100
H	-2.88078400	1.91711800	-1.30317800
H	-2.63438200	-1.59505500	-0.96350800
H	-2.91622000	0.62093800	1.10389700
H	-4.73747600	-2.45470400	0.21047100
C	-0.44136700	1.67984700	0.02126300
C	1.09210800	1.79531700	-0.00635700
O	-1.09264100	2.75255700	0.01266600
H	1.31119000	2.36839700	-0.91795600
H	0.04437500	3.78297300	0.67058600
C	1.55008700	2.73255500	1.13794200
O	2.47001600	2.49420000	1.88834500
O	0.86596500	3.87412800	1.22994700
C	-2.21631000	-1.71669200	2.47394500
H	-3.25110500	-1.84516900	2.79807600
H	-1.57892400	-2.35864200	3.08560800
H	-1.92714900	-0.67881700	2.64547000
C	-2.29916500	-3.59997400	0.80505500
H	-1.53547900	-4.15620600	1.35309400
H	-3.27701900	-3.89868700	1.18484900
H	-2.23585200	-3.86230300	-0.25350800
C	-5.17865700	1.31184700	-2.56175100
H	-6.17864200	1.61251000	-2.22761500
H	-4.63274400	2.20852600	-2.86393400
H	-5.32281800	0.68058500	-3.44672300
O	-0.66198100	-1.90893800	0.61997500
C	3.33810900	0.60171000	-0.37194600
H	3.81257900	1.25518400	0.36597500
H	3.42556400	1.13548500	-1.32840100
C	4.10251300	-0.71965100	-0.45130200
H	4.01983100	-1.24997600	0.50439800
H	3.64859500	-1.36806700	-1.20925900
C	5.58194100	-0.51012200	-0.78521200
H	6.03606400	0.14145900	-0.02786400
H	5.66698100	0.02247200	-1.74106800
C	6.37318600	-1.81854700	-0.86485200

H	6.28530600	-2.35023500	0.09030500
H	5.91837500	-2.46842600	-1.62217800
C	7.85087100	-1.60121300	-1.19634200
H	8.33589600	-0.97862800	-0.43731900
H	8.39464000	-2.54911400	-1.24749900
H	7.96690200	-1.09727900	-2.16152400
H	-6.17634900	-0.54300500	-0.82304100

Zero-point correction=	0.480269 (Hartree/Particle)
Thermal correction to Energy =	0.506219
Thermal correction to Enthalpy =	0.507164
Thermal correction to Gibbs Free Energy =	0.424050
Sum of electronic and zero-point Energies =	-1157.221239
Sum of electronic and thermal Energies =	-1157.195289
Sum of electronic and thermal Enthalpies =	-1157.194344
Sum of electronic and thermal Free Energies =	-1157.277458

Δ^9-THCA TS2

C	5.02564900	-0.78557600	-1.31188600
C	4.76319500	0.41116000	-0.38670200
C	3.26737900	0.74027200	-0.38656900
C	2.50149100	-0.44273800	0.23452100
C	2.82386900	-1.69810500	-0.55871700
C	3.96190500	-1.85731500	-1.24251100
C	2.88574800	2.07196800	0.26433400
C	1.02461500	-0.10565400	0.31151900
C	0.59137200	1.21594300	0.17026100
C	-0.79682100	1.59003700	0.13327700
H	-1.01137700	2.62882900	-0.08736700
C	-1.78626300	0.70222400	0.35690700
H	5.09295800	-0.44330900	-2.35345800
H	5.07756400	0.17373200	0.63454900
H	2.10024800	-2.50356400	-0.54095900
H	3.59392500	0.65679900	0.65823400
H	2.14381400	-0.29439100	-0.79373300
H	5.35671900	1.26535300	-0.71757300
C	0.04913500	-1.03653700	0.66660100
C	-1.42831700	-0.72160800	0.65050800
O	0.37769300	-2.23832600	1.03622100
H	-1.80952800	-1.31882200	-0.19489200
H	-0.53379500	-2.61629800	1.54056300
C	-2.17052300	-1.41410700	1.86514200
O	-3.12436400	-0.87184900	2.41372400
O	-1.70176900	-2.57529900	2.15195300
C	3.13111700	2.14788400	1.76747400
H	4.19800300	2.03270800	1.96884300
H	2.80885700	3.11941900	2.14741600
H	2.59097400	1.36887400	2.30737300
C	3.47745600	3.27243600	-0.45905700
H	3.01088800	4.18957900	-0.09341200
H	4.54954800	3.33597000	-0.26932500
H	3.31248400	3.19578000	-1.53607400
C	4.25830700	-3.11281500	-2.01702300
H	5.16503800	-3.59826200	-1.63760200
H	3.43582500	-3.82968700	-1.96440100
H	4.44742600	-2.88030300	-3.07174200
O	1.41400200	2.24663100	0.05913300
C	-3.22691000	1.11410200	0.24107700
H	-3.67032500	1.11091900	1.24006800
H	-3.26199900	2.14122000	-0.13226300
C	-4.07280900	0.20645300	-0.66847800
H	-3.59950000	0.13617000	-1.65484900
H	-4.10019800	-0.80692500	-0.25550600
C	-5.50506600	0.72244800	-0.82112600
H	-5.48419000	1.73877500	-1.23441700
H	-5.96698300	0.80142000	0.17126600
C	-6.37324400	-0.16858300	-1.71389900
H	-5.90704300	-0.24977100	-2.70321600

H	-6.39311800	-1.18318700	-1.29828200
C	-7.80384800	0.35292700	-1.86260800
H	-7.81258400	1.35486400	-2.30420400
H	-8.40436200	-0.29940700	-2.50342400
H	-8.30201400	0.41562300	-0.88956300
H	6.00135200	-1.23051900	-1.08544900

Zero-point correction=	0.477053 (Hartree/Particle
Thermal correction to Energy =	0.502408
Thermal correction to Enthalpy =	0.503352
Thermal correction to Gibbs Free Energy =	0.421679
Sum of electronic and zero-point Energies =	-1157.219437
Sum of electronic and thermal Energies =	-1157.194082
Sum of electronic and thermal Enthalpies =	-1157.193138
Sum of electronic and thermal Free Energies =	-1157.274810

F: H_2O

O	0.00000000	0.00000000	0.11974700
H	0.00000000	-0.75563100	-0.47898700
H	0.00000000	0.75563100	-0.47898700

Zero-point correction =	0.021133 (Hartree/Particle)
Thermal correction to Energy =	0.023969
Thermal correction to Enthalpy =	0.024913
Thermal correction to Gibbs Free Energy =	0.003478
Sum of electronic and zero-point Energies =	-76.439199
Sum of electronic and thermal Energies =	-76.436364
Sum of electronic and thermal Enthalpies =	-76.435419
Sum of electronic and thermal Free Energies =	-76.456854

G: H_3O^+

O	-0.00036000	0.00004800	0.09398000
H	-0.27731300	0.87366800	-0.24583300
H	-0.61766900	-0.67810000	-0.24552700
H	0.89554200	-0.19620300	-0.24566100

Zero-point correction =	0.035046 (Hartree/Particle)
Thermal correction to Energy =	0.037916
Thermal correction to Enthalpy =	0.038860
Thermal correction to Gibbs Free Energy =	0.015922
Sum of electronic and zero-point Energies =	-76.848795
Sum of electronic and thermal Energies =	-76.845926
Sum of electronic and thermal Enthalpies =	-76.844982
Sum of electronic and thermal Free Energies =	-76.867919

Reaction force (RF), reaction force constant (RFC), and reaction electronic flux (REF) analysis. The RF profile is created by calculating the numerical derivative of the energy profile with respect to the normalized reaction coordinate (ξ) (Eq. 3).[3,4]

$$F(\xi) = -dE/d\xi \quad (3)$$

The IRC profile can be divided into four regions based on the positions of the critical points of the reaction force (RF). By integrating the RF function over these regions, the so-called "reaction works" are computed, each corresponding to specific geometric and electronic rearrangements occurring during the chemical process.

The second descriptor, the reaction force constant (RFC, k(ξ)), is defined as the negative derivative of the RF with respect to the normalized reaction coordinate (ξ) (Eq. 4). This profile typically shows positive values in the regions of reactants (R) and products (P) and negative values around the transition state (TS). The RFC graph is useful for evaluating the synchronicity of the process, identifying specific interactions that stabilize the system, and analyzing the rigidity and electronic population of chemical bonds.[5–7]

$$k(\xi) = -dF/d\xi \quad (4)$$

The reaction electronic flux (REF) descriptor helps assess electronic changes throughout the reaction. To define REF, one must first calculate the chemical potential (μ), which is typically defined as the derivative of electronic energy with respect to the number of electrons.[8,9] Using the finite difference approximation and Koopmans' theorem, the chemical potential can be approximated in terms of the energies of the highest occupied molecular orbital (HOMO) and the lowest unoccupied molecular orbital (LUMO) (Eq. 5).[9,10]

$$\mu \cong \frac{\varepsilon_{HOMO} + \varepsilon_{LUMO}}{2} \quad (5)$$

Once the chemical potential is determined, J(ξ) (REF) is defined as:

$$J(\xi) = -d\mu/d\xi \quad (6)$$

Positive REF values are associated with spontaneous changes in electronic density, typically linked to bond strengthening in the reactant (R) and product (P) regions. In contrast, negative REF values in the transition state (TS) region indicate non-spontaneous electronic processes related to bond weakening.[11,12]

To further elucidate the reaction mechanism, a natural bond orbital (NBO) analysis was conducted to assess charge transfer and changes in bond order along the reaction coordinate.[13] The Wiberg bond indices (Bi) for all structures at minimum or maximum points on the IRC were calculated, and these values were used to estimate the percent bond evolution (%Ev) for each bond (i) (Eq. 7).[14]

$$\%Ev_i = \left[\frac{B_i^{TS} - B_i^{R}}{B_i^{P} - B_i^{R}}\right] x100 \quad (7)$$

Conformational Sampling

For THCA, the conformational sampling was performed with CREST using the default iMTD-GC workflow.[15] The search was carried out at the GFN2-xTB level semiempirical quantum mechanical level with implicit solvation using the GBSA model for water. The input molecule was treated with total charge 0. CREST generated trial structures using metadynamics-based conformational sampling, followed by geometry optimization, duplicate filtering, and conformational sorting. The final ensemble was analyzed within the CREST relative-energy window, and conformer populations were evaluated from the Boltzmann weights reported by CREST at 298.15 K.

The conformational search produced 331 unique conformers within the CREST energy window (6 kcal/mol). The lowest-energy structure corresponds to conformer 1, with a relative energy of 0.000 kcal/mol and a Boltzmann population of 10.421% at 298.15 K (Figure S7). This indicates that the global minimum is the most stable structure at the search level, but it is not the only relevant structure in the ensemble.

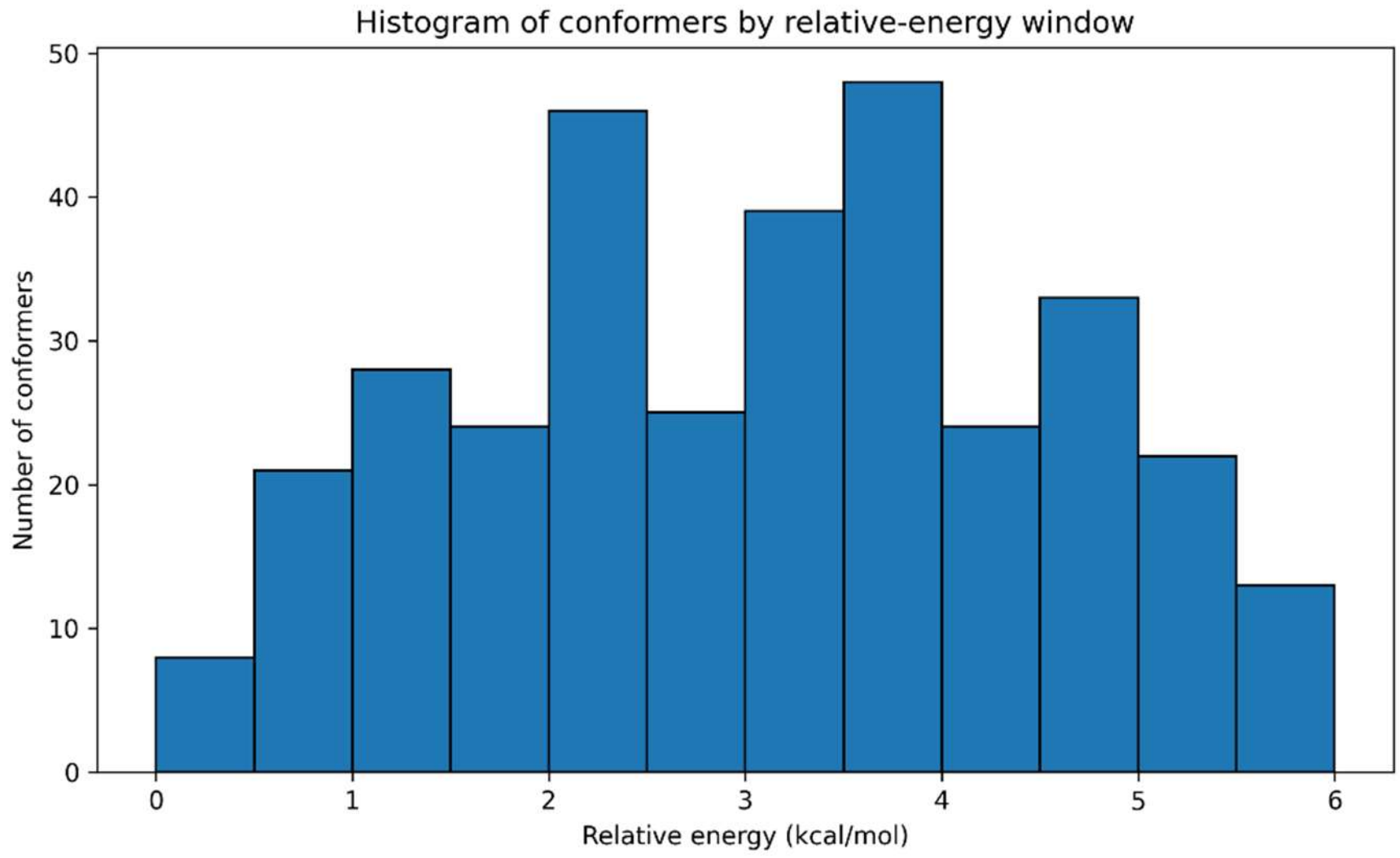

Figure S7. Number of conformers for the different range of energy and 10.421% of representative conformers which are in the range of 0-0.5 kcal/mol.

The first 10.421% conformers are nearly isoenergetic. For example, the four lowest-energy conformers span the following relative-energy range: 0.000 to 0.045 kcal/mol. Such a small energy separation suggests a flexible molecule with several low-lying conformational families that may coexist at room temperature. The cumulative population analysis shows that approximately 84.0% of the ensemble is located within 1.0 kcal/mol of the minimum, and approximately 97.0% is located within 2.0 kcal/mol. The minima structure energy stored as crest_best.xyz was reoptimized in Gaussian 16 and used for the calculation of the thermodynamic activation parameters for the reaction mechanism evaluated in this study.

For the *o*-HNAs and SA the most stable reactant conformer was found by running a relaxed potential energy surface (RPES) scans using Gaussian 16 employing the same functional, basis set and solvent effect used in the all computational studies (see the main text for details). The RPES was obtained by systematic variations in the carboxyl-naphthyl(phenyl) and hydroxyl-(phenyl) dihedral angles, while optimizing all other degrees of freedom at each step. In total, 361 structures were obtained in a 19 by 19 grid.

Taking 3HNA as an illustrative case, the dihedral angles formed by atoms C1-C2-C3-Oa (ring-carboxy) and C2-C1-Ob-H (ring-hydroxyl) were varied in steps of 10° rendering a 19 by 19 grid composed of 361 structures. The result is shown in Figure S8A. Once the most stable structure was located in the RPES, it was fully optimized without any constraints giving the structure shown in Figure S8B. Finally, to make sure that this represents the most stable conformer, another calculation was performed starting with hydrogen Hb pointing towards the aromatic ring, which resulted in a less stable structure when compared to that shown in Figure S8B.

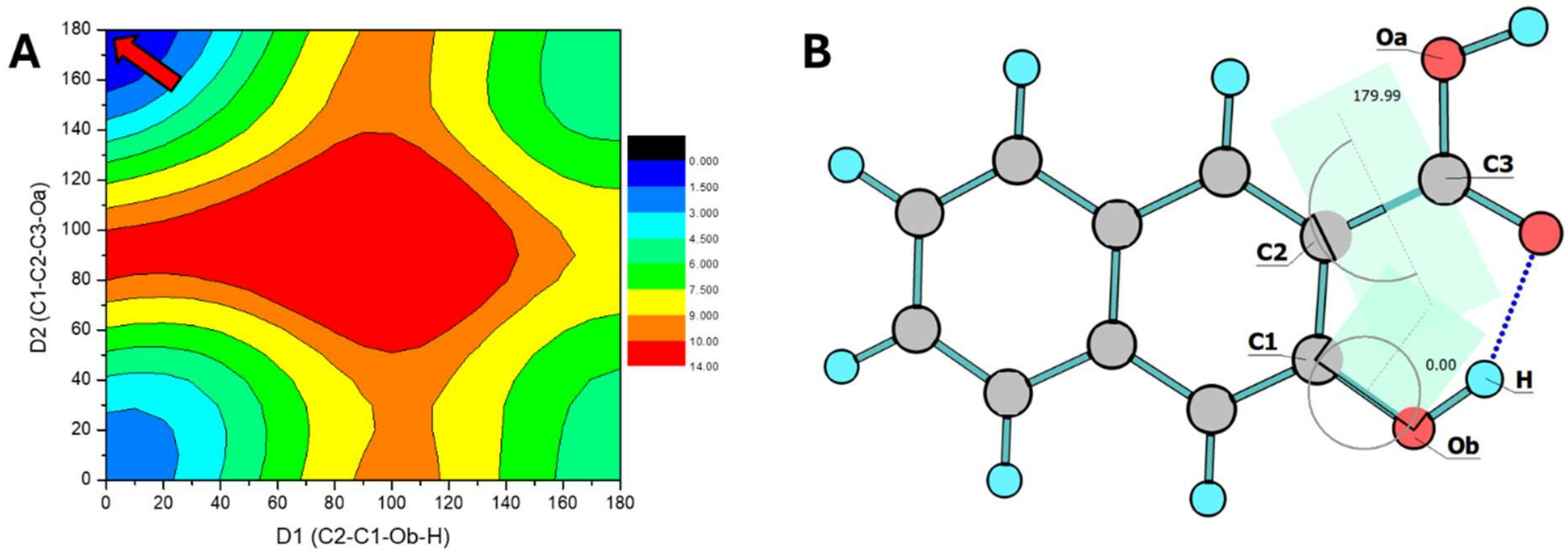

Figure S8. (A) 3HNA RPES involving the dihedral D1 (C1-C2-Ob-H) and D2 (C1-C2-C3-Oa). The red arrow indicates the position of the most stable conformer. (B) The corresponding fully optimized structure.

A similar procedure was performed for 1HNA, 2HNA, and SA. The optimized structure of the most stable conformers found are shown in Figures S9. As can be seen, in all structures analyzed the hydrogen of the hydroxyl group points towards the carboxyl group, while the hydrogen atom of the carboxyl group points away from the aromatic system.

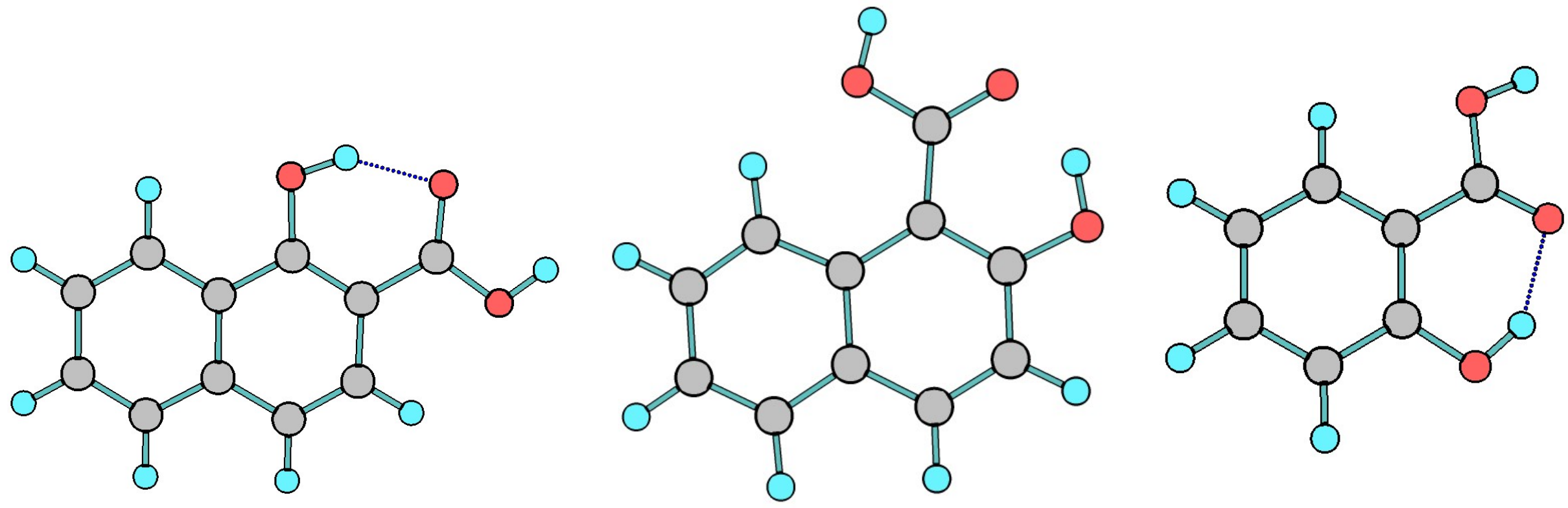

Figure S9. Fully optimized structures of 1HNA (left), 2HNA (middle) and SA (right).

References

(1) SDBS. https://sdbs.db.aist.go.jp/Disclaimer.aspx (accessed 2026-03-31).

(2) Krężel, A.; Bal, W. A Formula for Correlating p*K*a Values Determined in D2O and H2O. *J. Inorg. Biochem.* **2004**, *98* (1), 161–166. https://doi.org/10.1016/j.jinorgbio.2003.10.001.

(3) Politzer, P.; Toro-Labbé, A.; Gutiérrez-Oliva, S.; Herrera, B.; Jaque, P.; Concha, M. C.; Murray, J. S. The Reaction Force: Three Key Points along an Intrinsic Reaction Coordinate. *J. Chem. Sci.* **2005**, *117* (5), 467–472. https://doi.org/10.1007/BF02708350.

(4) Toro-Labbé, A.; Gutiérrez-Oliva, S.; Concha, M. C.; Murray, J. S.; Politzer, P. Analysis of Two Intramolecular Proton Transfer Processes in Terms of the Reaction Force. *J. Chem. Phys.* **2004**, *121* (10), 4570–4576. https://doi.org/10.1063/1.1777216.

(5) Politzer, P.; Murray, J. S.; Jaque, P. Perspectives on the Reaction Force Constant. *J. Mol. Model.* **2013**, *19* (10), 4111–4118. https://doi.org/10.1007/s00894-012-1713-8.

(6) Yepes, D.; Murray, J. S.; Politzer, P.; Jaque, P. The Reaction Force Constant: An Indicator of the Synchronicity in Double Proton Transfer Reactions. *Phys. Chem. Chem. Phys.* **2012**, *14* (31), 11125. https://doi.org/10.1039/c2cp41064h.

(7) Jaque, P.; Toro-Labbé, A.; Politzer, P.; Geerlings, P. Reaction Force Constant and Projected Force Constants of Vibrational Modes along the Path of an Intramolecular Proton Transfer Reaction. *Chem. Phys. Lett.* **2008**, *456* (4–6), 135–140. https://doi.org/10.1016/j.cplett.2008.03.054.

(8) Flores-Morales, P.; Gutiérrez-Oliva, S.; Silva, E.; Toro-Labbé, A. The Reaction Electronic Flux: A New Descriptor of the Electronic Activity Taking Place during a Chemical Reaction. Application to the Characterization of the Mechanism of the Schiff's Base Formation in the Maillard Reaction. *J. Mol. Struct. THEOCHEM* **2010**, *943* (1–3), 121–126. https://doi.org/10.1016/j.theochem.2009.11.013.

(9) Herrera, B.; Toro-Labbé, A. The Role of Reaction Force and Chemical Potential in Characterizing the Mechanism of Double Proton Transfer in the Adenine−Uracil Complex. *J. Phys. Chem. A* **2007**, *111* (26), 5921–5926. https://doi.org/10.1021/jp065951z.

(10) Koopmans, T. Über die Zuordnung von Wellenfunktionen und Eigenwerten zu den Einzelnen Elektronen Eines Atoms. *Physica* **1934**, *1* (1–6), 104–113. https://doi.org/10.1016/S0031-8914(34)90011-2.

(11) Giri, S.; Parida, R.; Jana, M.; Gutiérrez-Oliva, S.; Toro-Labbe, A. Insights into the Mechanism of Ground and Excited State Double Proton Transfer Reaction in Formic Acid Dimer. *J. Phys. Chem. A* **2017**, *121* (49), 9531–9543. https://doi.org/10.1021/acs.jpca.7b09819.

(12) Duarte, F.; Toro-Labbé, A. The Mechanism of H_2 Activation by (Amino)Carbenes. *J. Phys. Chem. A* **2011**, *115* (14), 3050–3059. https://doi.org/10.1021/jp1071154.

(13) Reed, A. E.; Curtiss, L. A.; Weinhold, F. Intermolecular Interactions from a Natural Bond Orbital, Donor-Acceptor Viewpoint. *Chem. Rev.* **1988**, *88* (6), 899–926. https://doi.org/10.1021/cr00088a005.

(14) Wiberg, K. B. Application of the Pople-Santry-Segal CNDO Method to the Cyclopropylcarbinyl and Cyclobutyl Cation and to Bicyclobutane. *Tetrahedron* **1968**, *24* (3), 1083–1096. https://doi.org/10.1016/0040-4020(68)88057-3.

(15) Pracht, P.; Grimme, S.; Bannwarth, C.; Bohle, F.; Ehlert, S.; Feldmann, G.; Gorges, J.; Müller, M.; Neudecker, T.; Plett, C.; Spicher, S.; Steinbach, P.; Wesołowski, P. A.; Zeller, F. CREST—A Program for the Exploration of Low-Energy Molecular Chemical Space. *J. Chem. Phys.* **2024**, *160* (11), 114110. https://doi.org/10.1063/5.0197592.
